# Structural and functional characterization of an achromatopsia-associated mutation in a phototransduction channel

**DOI:** 10.1038/s42003-022-03120-6

**Published:** 2022-03-01

**Authors:** Xiangdong Zheng, Huan Li, Zhengshan Hu, Deyuan Su, Jian Yang

**Affiliations:** Department of Biological Sciences, Columbia University, New York, NY 10027 USA

**Keywords:** Cryoelectron microscopy, Ion channels in the nervous system

## Abstract

Numerous missense mutations in cyclic nucleotide-gated (CNG) channels cause achromatopsia and retinitis pigmentosa, but the underlying pathogenic mechanisms are often unclear. We investigated the structural basis and molecular/cellular effects of R410W, an achromatopsia-associated, presumed loss-of-function mutation in human CNGA3. Cryo-EM structures of the *Caenorhabditis elegans* TAX-4 CNG channel carrying the analogous mutation, R421W, show that most apo channels are open. R421, located in the gating ring, interacts with the S4 segment in the closed state. R421W disrupts this interaction, destabilizes the closed state, and stabilizes the open state. CNGA3_R410W/CNGB3 and TAX4_R421W channels are spontaneously active without cGMP and induce cell death, suggesting cone degeneration triggered by spontaneous CNG channel activity as a possible cause of achromatopsia. Our study sheds new light on CNG channel allosteric gating, provides an impetus for a reevaluation of reported loss-of-function CNG channel missense disease mutations, and has implications for mutation-specific treatment of retinopathy.

## Abstract (web summary)

The R410W mutation in the cone photoreceptor CNG channel, linked to achromatopsia and assumed to be a loss-of-function variant, causes the channel to open spontaneously as revealed by cryo-EM, electrophysiology and calcium imaging, and is cytotoxic. This study calls for a multipronged evaluation/reevaluation of other inherited mutations associated with CNG channelopathy.

## Introduction

Vision starts with phototransduction in the retina. In vertebrates, phototransduction takes place in retinal rod and cone photoreceptors, where light activation of rhodopsin triggers a biochemical cascade that results in a decrease of the concentration of intracellular cyclic guanosine monophosphate (cGMP) and the closure of cGMP-activated CNG channels^1–4^. CNG channels open when bound with cGMP and conduct Na^+^ and Ca^2+^ ions into photoreceptor cells^1–4^. Vertebrate retinal CNG channels are heterotetrameric complexes of two different subunits, comprising of CNGA3 and CNGB3 subunits in cones and CNGA1 and CNGB1 subunits in rods^1,3–5^. As chemoelectrical transducers, CNG channels are essential for phototransduction and photoreceptor viability. Numerous inherited mutations in both rod and cone CNG channel genes have been associated with degenerative visual disorders such as retinitis pigmentosa and achromatopsia (ACHM)^1,3,5^. These mutations include frame-shift, deletion, insertion and missense mutations, and both loss-of-function (LOF) and gain-of-function (GOF) mutations have been reported^5–24^. Although the reasons of why and how frame-shift, deletion and insertion mutations cause diseases are usually evident, the pathogenic mechanisms of most missense mutations are generally more complicated and often obscure.

Recently, three-dimensional (3D) cryo-EM structures of eukaryotic CNG channels in apo closed state and cGMP-bound open state have been solved, including homotetrameric *C. elegans* TAX-4^25,26^, human CNGA1 channels^27^, and human CNGA1/CNGB1 channels^28^. These structures illustrate the conformational changes associated with ligand activation of the channels and provide a framework for testing the involvement and importance of various structural elements in this process. Importantly, these structures allow mapping of the 3D positions of most of the exonic disease-associated mutations (DAMs) and testing of how some of the DAMs alter channel structure and function. Up to date, no structures of CNG channels carrying DAMs have been reported, limiting our knowledge on the effects and mechanisms of DAMs on channel structure and gating. A structure-based investigation of DAMs may shed new light on CNG channelopathies and at the same time provide different perspectives into the molecular mechanisms of CNG channel allosteric gating.

More than 150 and 125 mutations in the *CNGA3* and *CNGB3* genes, respectively, have been linked to ACHM^1,5–7,15,17,29–37^, an autosomal recessive disease producing incomplete or complete loss of cone photoreceptor function, with an estimated prevalence of 1:30,000^5,38^. ACHM patients are characterized by partial or total loss of color vision, photophobia, nystagmus, and decreased visual acuity^7^. Currently, there is no cure for ACHM, but new treatment strategies are being developed, including adeno-associated virus-based gene supplementation therapy, in which the WT CNGA3 subunit is overexpressed in diseased cone photoreceptors^5,39–43^. Phase I and II clinical trials of this therapy are in progress^5^. Elucidating how ACHM-associated mutations (AAMs) alter CNG channel structure and function is valuable for devising proper therapeutic treatments for ACHM. Most AAMs in CNGA3 are missense mutations^5–7^, and most of the previously characterized missense AAMs have been reported to cause a LOF phenotype at the molecular and cellular level, such as aberrant protein turnover, misfolding, mislocalization, defective gating, and impaired posttranslational modification^8–19^. In one study, 32 of the 39 missense AAMs in CNGA3 have been found to produce little or no whole-cell currents when expressed in HEK 293 cells and are considered as LOF mutations^10^.

**Fig. 1:**
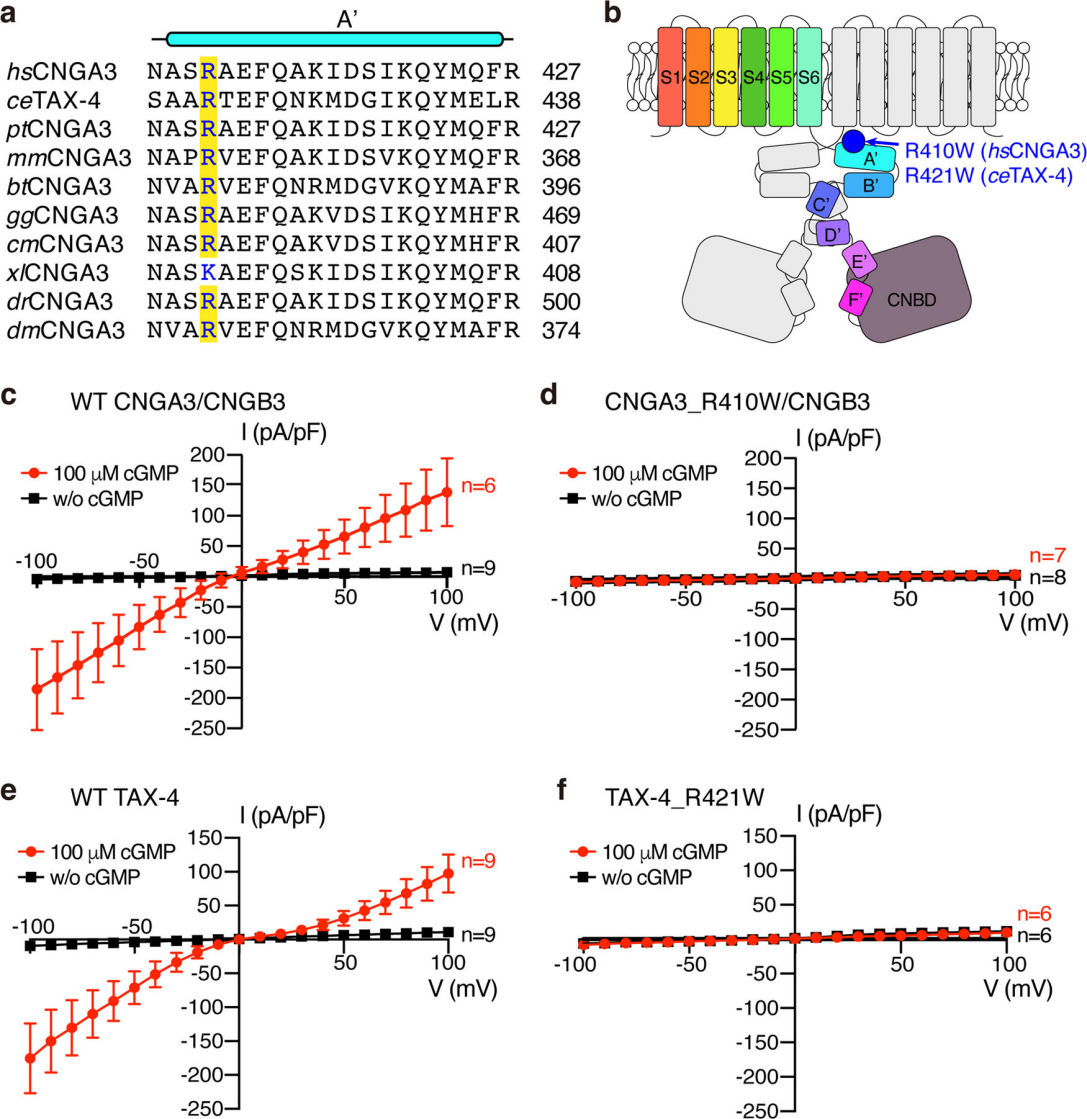
Whole-cell recordings of WT and mutant channels. **a** Amino acid sequence alignment of A’ α helix from different species. Species abbreviation: *hs* *Homo sapiens*, *ce* *Caenorhabditis elegans*, *pt* *Pan troglodytes*, *mm* *Mus musculus*, *bt* *Bos taurus*, *gg* *Gallus gallus*, *cm* *Chelonia mydas*, *xl* *Xenopus laevis*, *dr* *Danio rerio*, *dm* *Drosophila melanogaster*. **b** Cartoon depiction of CNG channel subunits and location of R410W in CNGA3 and R421W in TAX-4. **c**–**f** Current-voltage (I-V) relationships of HEK 293T cells transfected with WT CNGA3/CNGB3 (**c**), CNGA3_R410W/CNGB3 (**d**), WT TAX-4 (**e**), and TAX-4_R421W (**f**). Data are presented as mean ± SD. *n* number of cells.

Taking advantage of the ability to obtain high-resolution 3D structures of full-length TAX-4 CNG channels in both apo and cGMP-bound states, we reevaluated and investigated the structural and cellular effects of R410W, an AAM in human CNGA3, and of R421W, the analogous mutation in TAX-4. R410 is conserved in eukaryotic CNGA3 (Fig. 1a) and is located in the gating ring (Fig. 1b). The R410W mutation produces complete color blindness^15,31^ and is reportedly a LOF mutation^10^. Our results show that R421W opens TAX-4 in the absence of cGMP, that R421W/R410W increases the spontaneous activity of TAX-4 and human CNGA3/CNGB3 channels, and that expression of the R410W mutant channel causes cell death. As proper characterization of DAMs may be instrumental for devising suitable therapeutic treatments of mutation-specific visual disorders, our findings call for a reevaluation of some previously characterized missense DAMs.

## Results

### R410W is an apparent LOF mutation

R410 is conserved in CNGA3 from nematode to human (Fig. 1a). Structurally, it is located in the A’ α helix of the gating ring, a crucial structural element sandwiched between the transmembrane domain (TMD) and the cyclic nucleotide-binding domain (CNBD) (Fig. 1b). The gating ring transmits cGMP binding/unbinding-triggered conformational changes in the CNBD to the TMD, where a cavity gate formed by two hydrophobic residues in S6 opens and closes^25–28^. R410 is four amino acids away from the C-terminal end of S6 and interacts with amino acids in other regions involved in ligand gating in both open and closed states, as will be described in Discussion. We thus focused on R410 and hypothesized that R410 plays a critical role in cGMP activation and its mutation likely impairs gating.

We first examined whether R410W affects cGMP activation of human CNGA3/CNGB3 channels transiently expressed in HEK 293T cells. Cells transfected with wild-type (WT) channels (cotransfected with enhanced GFP) produced robust currents, induced by 100 μM cGMP in the whole-cell recording pipette (Fig. 1c), but cells transfected with R410W mutant channels had no currents, as mock-transfected cells did (Fig. 1d). We also tested the effect of the equivalent mutation on TAX-4 activity and found that cells transfected with TAX-4_R421W mutant channels did not produce cGMP-activated currents (Fig. 1e, f). These results are consistent with a previous report^10^ and with the interpretation that R410W causes a loss of channel function. However, as will be demonstrated later, this interpretation needs to be revised.

### Mutant TAX-4 can be activated by cGMP

To better understand how the R410W mutation alters channel structure and function, and because we have already obtained closed- and open-state cryo-EM structures of TAX-4^25,26^, we proceeded to determine the cryo-EM structures of TAX-4_R421W in apo and cGMP-bound states. Details of protein purification, cryo-EM data collection and processing and atomic model refinement and validation can be found in Table 1 and relevant Supplementary figures (Supplementary Figs. 1–5) and in Methods.

**Table 1:** Cryo-EM data collection, refinement and validation statistics.

	cGMP-bound open state (EMD-24113, PDB 7N15)	Apo open state (EMD-24115, PDB 7N17)	Apo closed state (EMD-24114, PDB 7N16)
Data collection and processing			
Magnification	×105,000	×105,000	
Voltage (kV)	300	300	
Electron exposure (e−/Å^2^)	69.7	58	
Defocus range (μm)	−0.9–−1.5	−0.9–−1.5	
Pixel size (Å)	0.83	0.83	
Initial particle images (no.)	3,988,515	1,679,420	
Final particle images (no.)	284,615	100,919	66,523
Map resolution (Å)	2.9	3.1	3.2
FSC threshold	0.143	0.143	0.143
Map resolution range (Å)	2.4–3.2	2.4–4.0	2.4–4.0
Symmetry imposed	*C*4	*C*4	*C*4
Refinement			
Initial model used (PDB code)	6WEK	6WEK	6WEJ
Model resolution (Å)	2.96	3.15	3.32
FSC threshold	0.143	0.143	0.143
Map sharpening *B* factor (Å^2^)	−143	−127	−116
Model composition			
Non-hydrogen atoms	17,800	17,596	17,357
Protein residues	2080	2068	2056
Ligands (cGMP/lipids/Na^+^)	4/24/0	0/24/0	0/16/1
* B* factors (Å^2^)			
Protein	30.83	82.87	92.94
Ligand	30.31	55.98	71.78
R.m.s. deviations			
Bond lengths (Å)	0.007	0.009	0.008
Bond angles (°)	0.939	1.118	0.984
Validation			
MolProbity score	1.27	1.38	1.62
Clashscore	4.21	3.44	4.59
Poor rotamers (%)	0.43	1.28	0.86
Ramachandran plot			
Favored (%)	97.68	97.09	94.31
Allowed (%)	2.32	2.91	5.69
Disallowed (%)	0	0	0

**Fig. 2:**
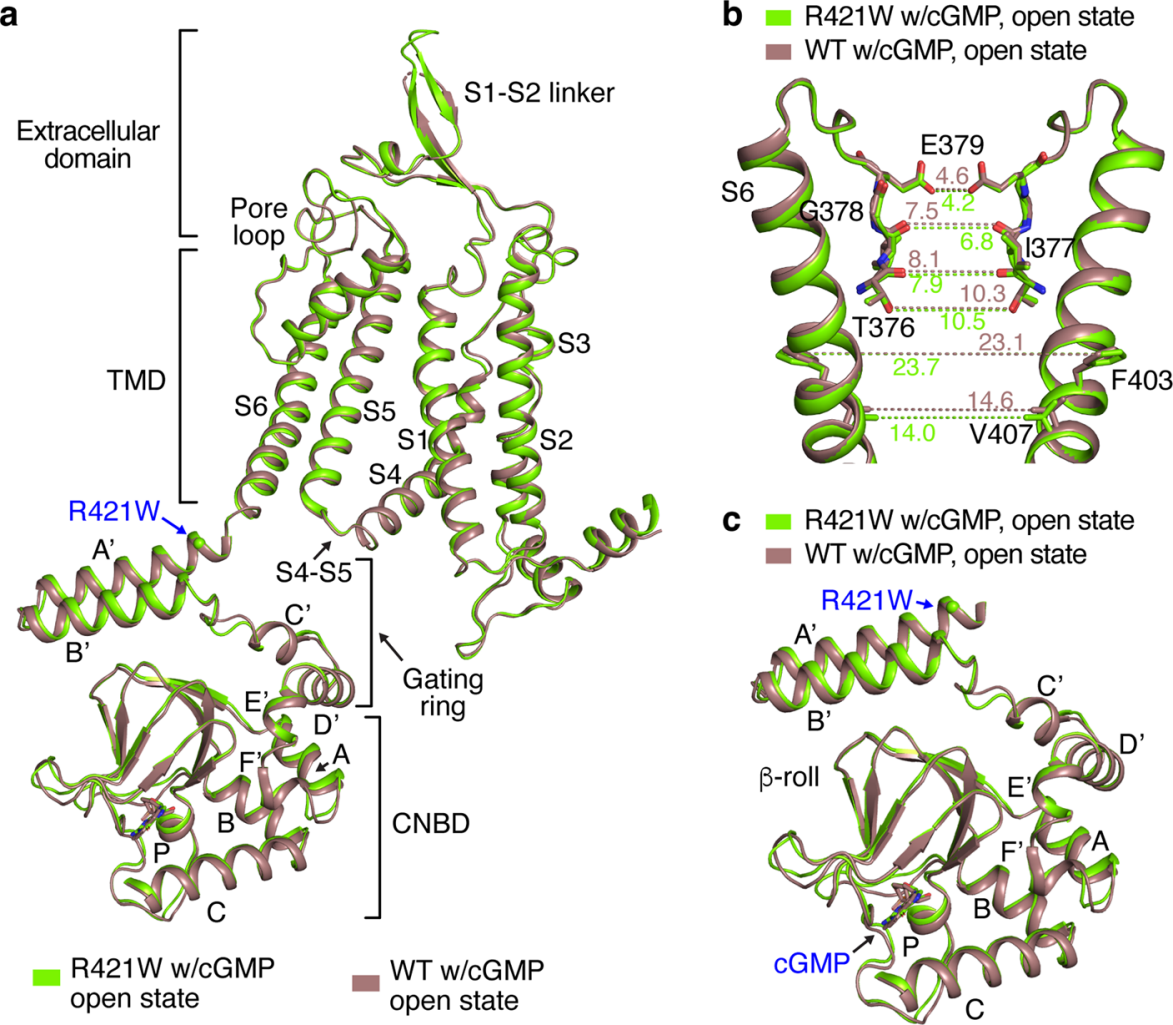
Structure of cGMP-bound TAX-4_R421W. **a** Superposition of protomer structures of cGMP-bound WT (PDB ID: 6WEK) and mutant TAX-4. **b** Comparison of the SF (formed by T376, I377, G378 and E379) and central cavity (below the SF). Distances between two diagonally opposed subunits are indicated. **c** Comparison of the CNBD and C-linker.

Given that the R421W mutation appears to greatly attenuate cGMP activation of TAX-4 (Fig. 1e, f), we at first hypothesized that either TAX-4_R421W could not bind cGMP or cGMP-bound TAX-4_R421W would be closed due to mutation-induced defective coupling of cGMP binding and gate opening. To our surprise, cGMP-bound TAX-4_R421W is open and its structure (determined at 2.9 Å resolution with *C*4 symmetry) is identical to the previously reported structure of cGMP-bound WT TAX-4^25^ (Fig. 2). TAX-4 can be arbitrarily divided into four structural layers from the outside to the inside, including an extracellular domain, the TMD (made up of S1–S6 helices), the C-linker (made up of A’–F’ helices, of which A’–D’ forms the gating ring), and the CNBD^25,26^ (Fig. 2a). Comparison of mutant and WT channel structures (Fig. 2a) shows an r.m.s.d. of 0.62 Å when all visible amino acids are included. The hydrophobic cavity gate formed by F403 and V407 of S6 opens to the same extent in the mutant channel as in the WT channel (Fig. 2b), and the CNBD and gating ring in both channels have the same ligand-bound conformation (Fig. 2d). These results suggest that the R421W mutation does not markedly affect the process of cGMP activation of TAX-4.

### Mutant TAX-4 is open without cGMP

**Fig. 3:**
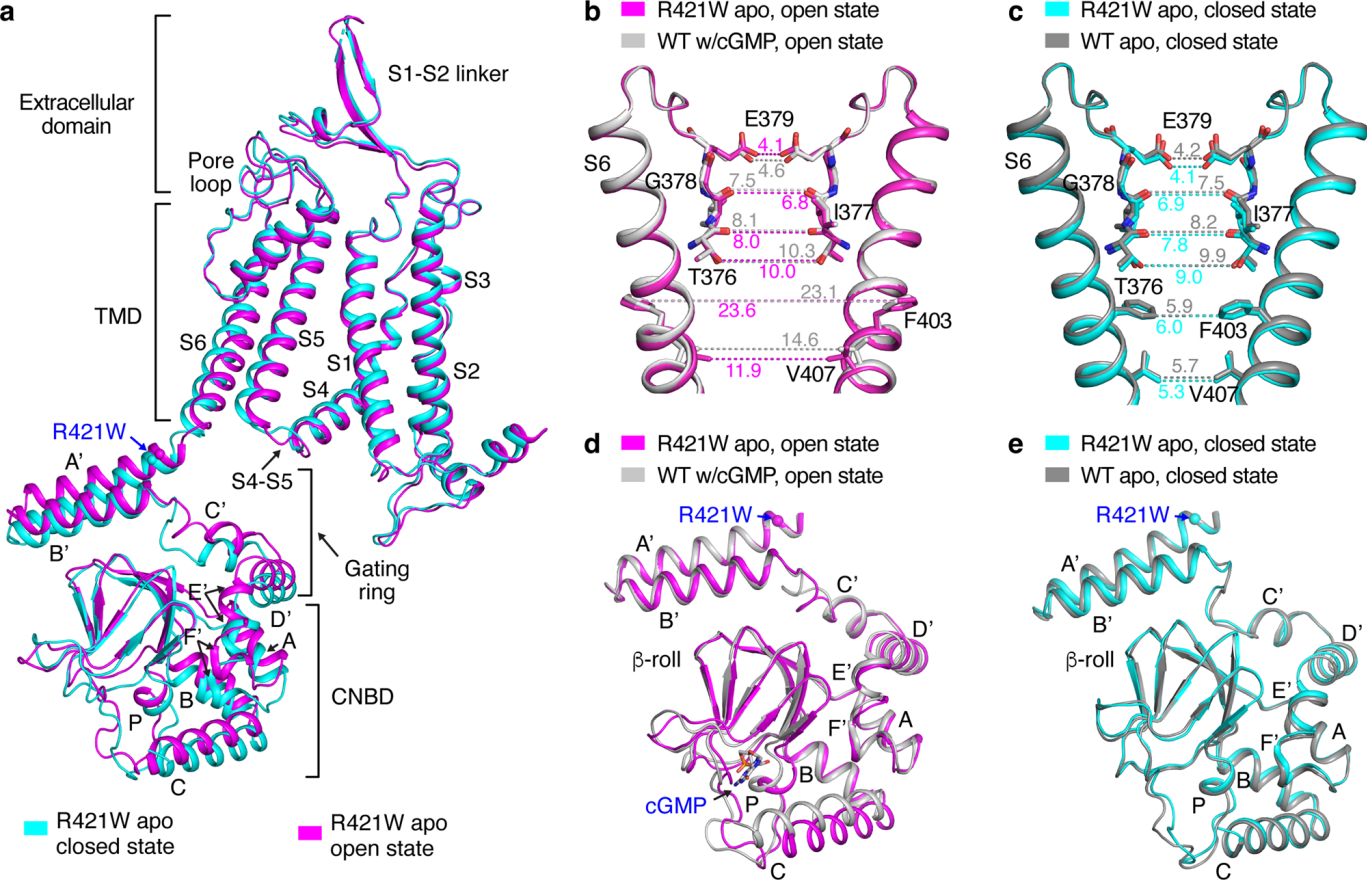
Structures of apo TAX-4_R421W. **a** Superposition of protomer structures of the closed and open states of apo TAX-4_R421W. **b** Comparison of the SF and central cavity in the open states of apo TAX-4_R421W and cGMP-bound WT TAX-4 (PDB ID: 6WEK). **c** Comparison of the SF and central cavity in the closed states of apo TAX-4_R421W and apo WT TAX-4 (PDB ID: 6WEJ). **d** Comparison of the CNBD and C-linker in the open states of apo TAX-4_R421W and cGMP-bound WT TAX-4. The C helix of apo WT TAX-4 is also shown. **e** Comparison of the CNBD and C-linker of the closed states of apo TAX-4_R421W and apo WT TAX-4.

What then does the R421W mutation do to the channel structurally? Since this mutation did not affect the cGMP-bound open-state structure, we hypothesized that it must have changed the apo closed-state structure. Surprisingly, consensus 3D refinement of apo TAX-4_R421W cryo-EM single particles revealed substantial heterogeneity and suggested the existence of channels in both closed and open states (Supplementary Fig. 2). Further processing with an S6-gating ring-CNBD mask and focused 3D classification showed that indeed apo TAX-4_R421W existed not only in the closed state but also in an open state; the two structures were determined at 3.2 and 3.1 Å resolution, respectively, with *C*4 symmetry (Fig. 3 and Supplementary Fig. 2c). Remarkably, more channels are in the open state than in the closed state (Supplementary Fig. 2c). To ensure that the apo open-state of TAX-4_R421W was not caused by targeted masking and focused 3D classification, we processed an apo WT TAX-4 dataset using the same procedures and mask, which produced only the apo closed state (Supplementary Fig. 6). This result indicates that the R421W mutation is the only cause for the mutant channel to open in absence of cGMP.

The open-state structure of apo TAX-4_R421W is identical to the cGMP-bound open-state structure of WT TAX-4 in virtually every part of the channel (Fig. 3a), including the selectivity filter (SF) (Fig. 3b), the cavity gate (Fig. 3b), the C-linker (Fig. 3d), and even most part of the CNBD (Fig. 3d). The structures of the C helix and some loops in the CNBD and the loop between helices B’ and C’ of the gating ring are different to some extent (Fig. 3d). Excluding these divergent regions, comparison of the open-state structures of apo TAX-4_R421W and cGMP-bound WT TAX-4 shows an r.m.s.d. of 0.48 Å. The closed-state structures of apo TAX-4_R421W and apo WT TAX-4 are identical, with an r.m.s.d. of 0.50 Å when all visible amino acids are compared (Fig. 3c, e). These results indicate that the R421W mutation greatly destabilizes the closed state and stabilizes the open state, rendering a majority of the mutant channels open in the absence of an activating ligand.

### Mutant channels are spontaneously active without cGMP

**Fig. 4:**
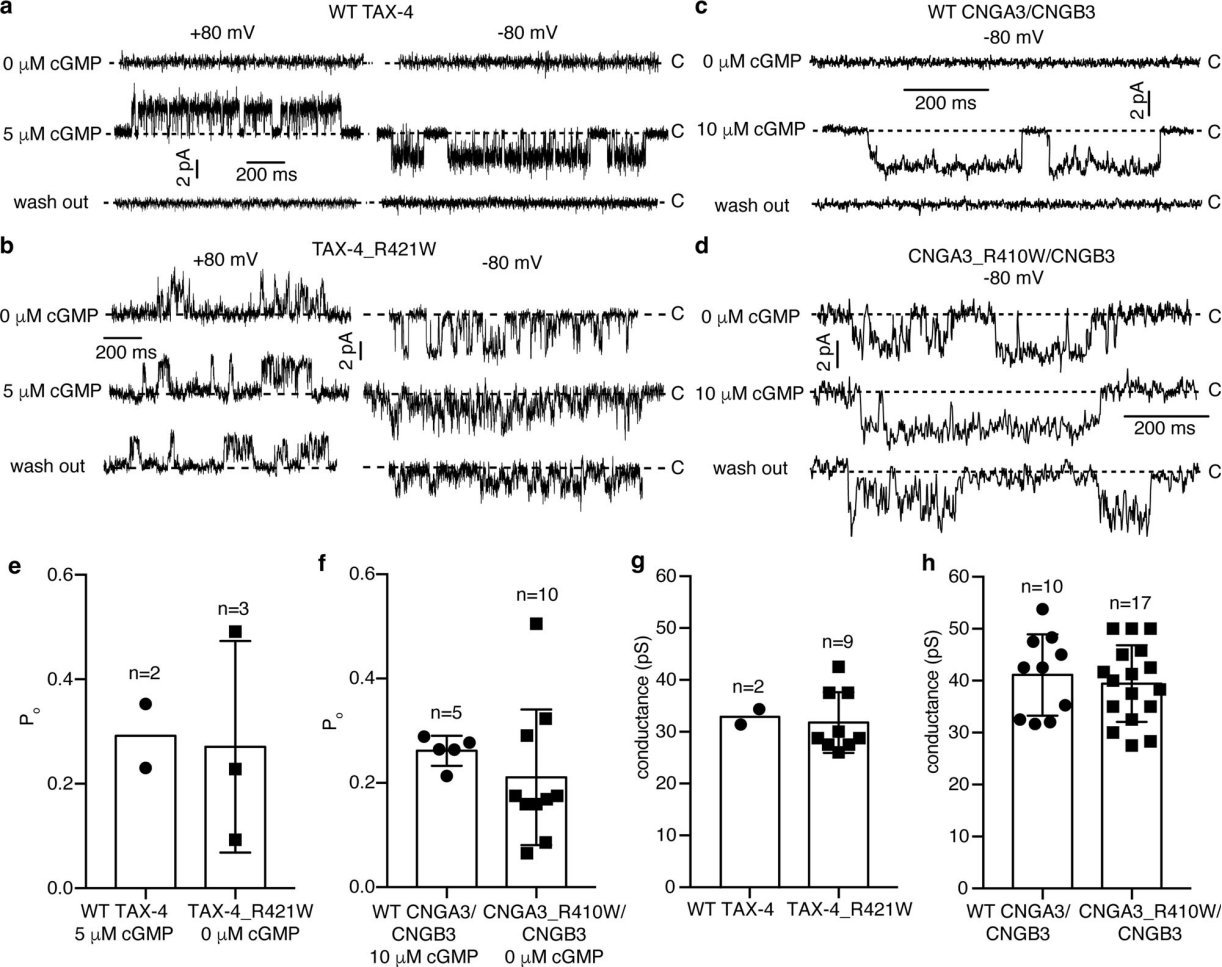
Single-channel recording of purified channels. **a**–**d** Single-channel currents of the indicate channels reconstituted in liposomes. Currents were recorded at the indicated membrane potentials in the absence or presence of 5 μM or 10 μM cGMP in the bath. Dashed lines mark the closed baseline. **e**–**h** Single-channel open probability (P_o_) (**e**, **f**) and conductance (**g**, **h**) of the indicated channels. Each point represents a different patch. Currents of WT TAX-4 and CNGA3/CNGB3 channels were evoked by 5 μM or 10 μM cGMP, and currents of the mutant channels were spontaneous openings in the absence of cGMP. Data are presented as mean ± SD. *n* number of patches. Patches with high noise levels were not included in the P_o_ analysis.

The observation of apo TAX-4_R421W in an open state suggests that CNG channels carrying this mutation would conduct currents in the absence of cGMP. Because TAX-4_R421W and CNGA3_R410W/CNGB3 channels exhibited neither cGMP-activated currents nor discernible basal currents in whole-cell recordings (Fig. 1d, f) (a possible reason for these observations is presented in “Discussion”), we tested this prediction on TAX-4 and CNGA3/CNGB3 channels in two other ways. In the first test, we reconstituted purified WT and mutant channel proteins into liposomes and recorded their single-channel activities in the absence and presence of cGMP. None of the 16 patches of WT TAX-4 shows spontaneous activities, including the 2 patches that display cGMP-activated currents, as exemplified by the patch in Fig. 4a. In contrast, of the 58 patches of TAX-4_R421W, 9 show spontaneous activities, as represented by the patch in Fig. 4b. Similarly, none of the 42 recordings of WT CNGA3/CNGB3 shows activities in the absence of cGMP, including the 10 patches that show cGMP-induced currents, as exemplified by the patch in Fig. 4c. In contrast, of the 60 recordings of CNGA3_R410W/CNGB3, 17 patches show spontaneous currents in the absence of cGMP, as represented by the patch in Fig. 4d.

It is noticeable that the openings of WT and mutant channels vary markedly in amplitude and kinetics. For example, the spontaneous open probability of the mutant channels varies from 0.09 to 0.49 for TAX-4_R421W and from 0.09 to 0.5 for CNGA3_R410W/CNGB3 (Fig. 4e, f). This is probably a result of the harsh treatment that the channel proteins endured during liposome reconstitution. Thus, the liposome recordings may be inadequate for making quantitative comparisons of spontaneous and cGMP-induced single-channel currents. Nevertheless, the single-channel conductance of TAX-4_R421W, calculated from its spontaneous openings, is 31.8 ± 5.9 pS (Fig. 4g), which is similar to the reported 32 pS for WT TAX-4^44^. The single-channel conductance of CNGA3_R410W/CNGB3, obtained from its spontaneous openings, is 39.4 ± 7.4 pS (Fig. 4h). This is similar to the 41.1 ± 7.8 pS we obtained (Fig. 4h) and the 41-42 pS reported for WT CNGA3/CNGB3^21,45^. Moreover, the cGMP-induced currents of WT CNGA3/CNGB3 and the spontaneous currents of CNGA3_R410W/CNGB3 are inhibited by 100 μM L-*cis*-diltiazem (DTZ), a blocker of native cone CNG channels and heterologously expressed CNGA3/CNGB3 channels (Supplementary Fig. 7). These results together indicate that the spontaneous openings of the mutant channels are indeed bona fide channel activities produced by CNGA3/CNGB3 heteromeric channels.

**Fig. 5:**
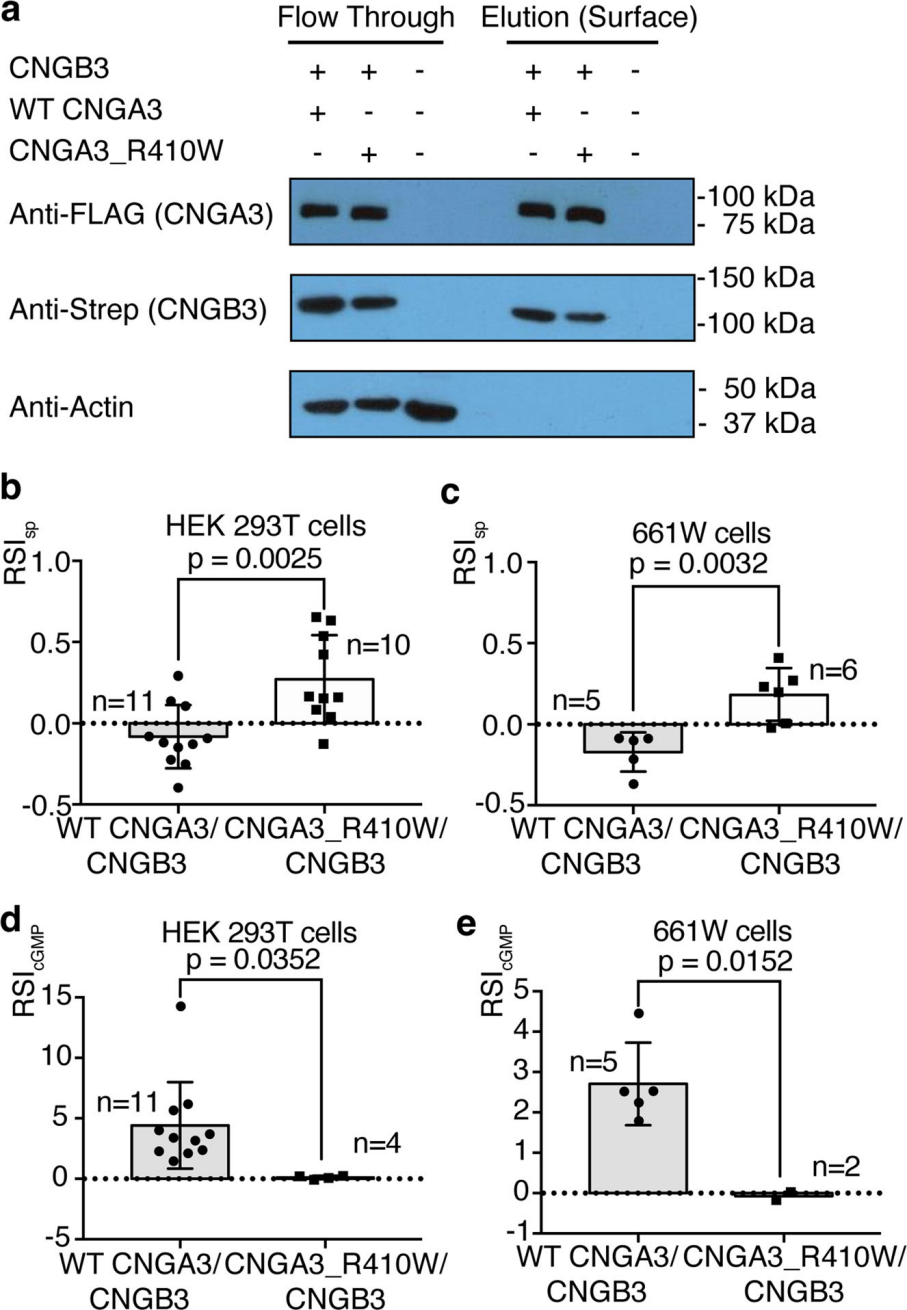
Spontaneous activity of surface mutant CNGA3/CNGB3 channels revealed by Ca2+ imaging. **a** Western blot with the indicated antibodies of transiently expressed WT and mutant CNGA3 and CNGB3 subunits from one preparation of surface biotinylation. The elution fraction was collected from the neutravidin-agarose beads and represents cell surface proteins. Flow through contained all the proteins that did not bind to the neutravidin-agarose beads, including cytoplasmic and organellar proteins. Anti-actin was used as a loading control and an indicator of cell integrity and biotinylation specificity. Similar results were observed in two other preparations. **b**, **c** Ca^2+^ imaging of HEK 293T and 661W cells transfected with WT and mutant human CNGA3/CNGB3 channels. The cells were first incubated with 2 mM CaCl_2_ and 100 µM diltiazem (DTZ) without cGMP. Then CaCl_2_ and DTZ were washed off and the cells were treated with 2 mM CaCl_2_. The normalized fluorescent signal increase upon switching from Ca^2+^ + DTZ to Ca^2+^-only is defined as spontaneous Relative Signal Increase (RSI_sp_). Each point represents one well of cells from one of four to six different cultures. **d**, **e** Ca^2+^ imaging of HEK 293T and 661W cells transfected with the indicated channels, treated with 100 μM CPT-cGMP. The normalized fluorescent signal increase upon switching from 2 mM CaCl_2_ to 2 mM CaCl_2_ and 100 μM CPT-cGMP is defined as RSI_cGMP_. Each point represents one well of cells from one of four to six different cultures. Data are presented as mean ± SD. Statistical significance was evaluated with Student’s *t* test.

In the second test, we examined by Ca^2+^ imaging the basal activities of surface WT and mutant CNGA3/CNGB3 channels heterologously expressed in HEK 293T cells and 661W cells, which are immortalized cone photoreceptor cells derived from mouse^46^ and have been widely used as a model for studying photoreceptor function, signaling, pathology and cell biology (see e.g.^47–49^). Cone photoreceptor CNG channels are highly permeable to Ca^2+ ^^50^; thus, intracellular Ca^2+^ levels can be used as a proxy indicator of the activity of CNGA3/CNGB3 channels at the plasma membrane. Surface biotinylation shows that CNGA3_R410W and CNGB3 are expressed at the plasma membrane (Fig. 5a). The intracellular Ca^2+^ concentration did not change noticeably upon switching the extracellular bath from a solution containing 2 mM CaCl_2_ and 100 µM DTZ to a solution containing 2 mM CaCl_2_ in HEK 293T and 661W cells transfected with WT CNGA3/CNGB3, but increased markedly in cells transfected with CNGA3_R410W/CNGB3 (Fig. 5b, c and Supplementary Figs. 8–10). These increases are likely an underestimate of the actual increases considering that the transfection efficiency was <25% (Supplementary Figs. 8 and 9) and that many transfected cells, especially those strongly express the mutant channels, are likely dead (see below). These results indicate that CNGA3_R410W/CNGB3 mutant channels at the plasma membrane are spontaneously open in the absence of cGMP in unstimulated cells, including cone photoreceptors.

As expected, application of a membrane-permeable cGMP, CPT-cGMP, produced a large increase in intracellular Ca^2+^ concentration in cells transfected with WT CNGA3/CNGB3 (Fig. 5d, e). In contrast, CPT-cGMP had little effect in cells transfected with CNGA3_R410W/CNGB3 (Fig. 5d, e). This observation is consistent with the lack of whole-cell currents in cells transfected with the mutant channel (Fig. 1d) and with the observation below that this mutant channel is cytotoxic.

### The mutant channel is cytotoxic

**Fig. 6:**
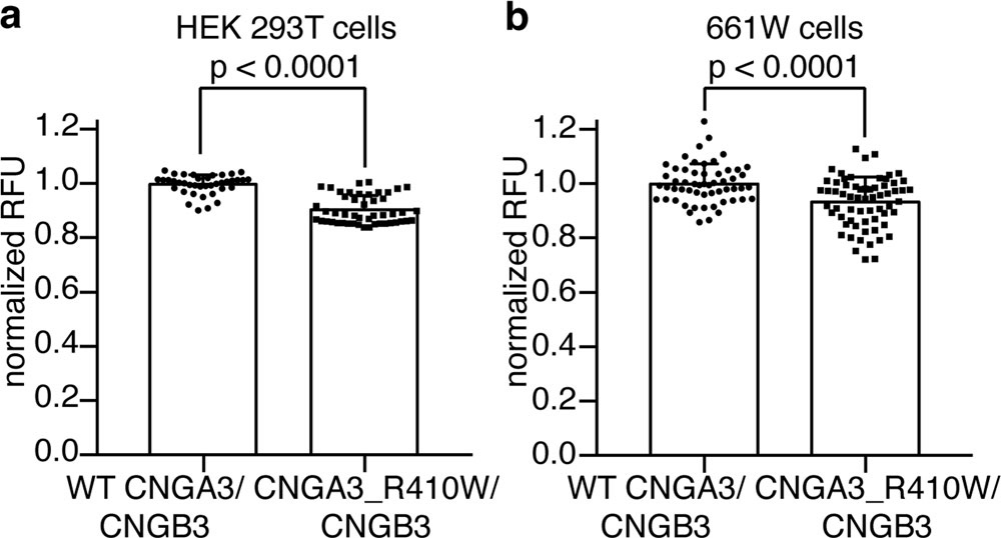
The mutant channel causes cell death. Cell viability of HEK 293T (**a**) and 661W cells (**b**) 48 h after transfection with WT or mutant CNGA3/CNGB3. Each point represents one well of cells from one of six different cultures. Data are presented as mean ± SD. Statistical significance was evaluated with Student’s *t* test.

We next examined whether the spontaneous basal activity of CNGA3_R410W/CNGB3 mutant channels is toxic to cells. The Resazurin assay^51^ was used to compare viability of HEK 293T and 661W cells transfected with WT or mutant CNGA3/CNGB3. The results show that 48 h after transfection cells transfected with the mutant channel have a significantly reduced viability in comparison to cells transfected with the WT channel (Fig. 6). This effect is likely underestimated because most of the cells examined were untransfected. We further tested whether blocking CNGA3/CNGB3 channels with DTZ has a protective effect, but unfortunately, long-term incubation of HEK 293T cells with 10 μM DTZ appears to be toxic on its own (Supplementary Fig. 11). Nevertheless, comparisons of the results from strictly parallel experiments suggest that the mutant channel is toxic to cells, most likely due to its spontaneous activity.

## Discussion

In this study we present structures of a eukaryotic homomeric CNG channel carrying a disease mutation in apo and cGMP-bound states and demonstrate that a single disease mutation can dramatically alter allosteric gating. We find that R410W in CNGA3, a previously reported LOF DAM^10^, in fact increases the activity of the mutant channel. At the molecular level, a LOF of an ion channel is usually defined by a reduction or loss of currents, often caused by defective surface expression or gating (see e.g.^52–54^). Our work points to a less-considered and less-tested possibility for a loss of ion channel currents in cells: a mutant channel becomes spontaneously active and is thus toxic to cells, and cells with high levels of surface channels die during cell culture. As a result, patch-clamp recordings are biased toward cells containing just a cotransfected fluorescent marker or cells with mainly intracellular channels, and these cells produce little or no currents. Our results suggest that this is the case for the R410W mutation.

At first glance, there appears to be a contradiction between the results of whole-cell recording (a lack of basal whole-cell current, Fig. 1d) and of Ca^2+^ imaging (an increase of Ca^2+^ influx, Fig. 5b, c) of CNGA3_R410W/CNGB3. But a key difference in these two experiments may account for the apparent discrepancy. Whole-cell recording measures currents from individual cells, and its sensitivity makes it difficult to distinguish very small basal currents (<100–200 pA) produced by spontaneously open channels from leak or poor seal. On the other hand, Ca^2+^ imaging measures the cumulative influx of Ca^2+^ from hundreds of cells. Thus, although the spontaneously active mutant channels in the surviving cells may not generate large enough currents that can be detected by whole-cell recording, they generate large enough Ca^2+^ signals that can be measured by imaging hundreds of cells together. The channels detected by surface biotinylation in our study also likely come from the surviving cells that express low levels of mutant channels on the plasma membrane.

Based on our findings, and in light of a previous report that channel hyperactivity and Ca^2+^ overload cause death of 661W cells expressing a GOF DAM (F525N) in CNGB3^23^, we propose the following scenario for how R410W produces its disease phenotype: The R410W mutation causes CNGA3/CNGB3 channels at the plasma membrane to open in the absence of cGMP and thus allow Na^+^ and Ca^2+^ ions to flow continuously into cone photoreceptors at all times. This continuous cation influx leads to Ca^2+^ overload and dissipation of the membrane potential. Meanwhile, spontaneously active mutant channels trapped in the ER and other intracellular organelles produce a continuous release of Ca^2+^ as well as degradation of organellar ionic gradients. Together, the spontaneous activities of surface and intracellular mutant channels contribute to cause the degeneration of cone photoreceptors.

At the molecular level, R410W can be considered as a GOF mutation as it renders the mutant channel spontaneously active. Several other DAMs in CNGA3 and CNGB3 have been shown to be GOF mutations^8,20–24^. The underlying molecular changes include increased ligand sensitivity^8,20–24^ and increased ligand efficacy^8,21^. On the other hand, some DAMs produce more complex or even mixed GOF and LOF effects, including increased ligand sensitivity, altered pore properties, decreased surface expression, and altered regulation by phosphoinositides^21,24^. It is possible that R410W may also cause other changes in the CNGA3/CNGB3 channel that contribute to its disease phenotype, such as misprocessing, mislocalization and misregulation. These possibilities remain to be investigated.

**Fig. 7:**
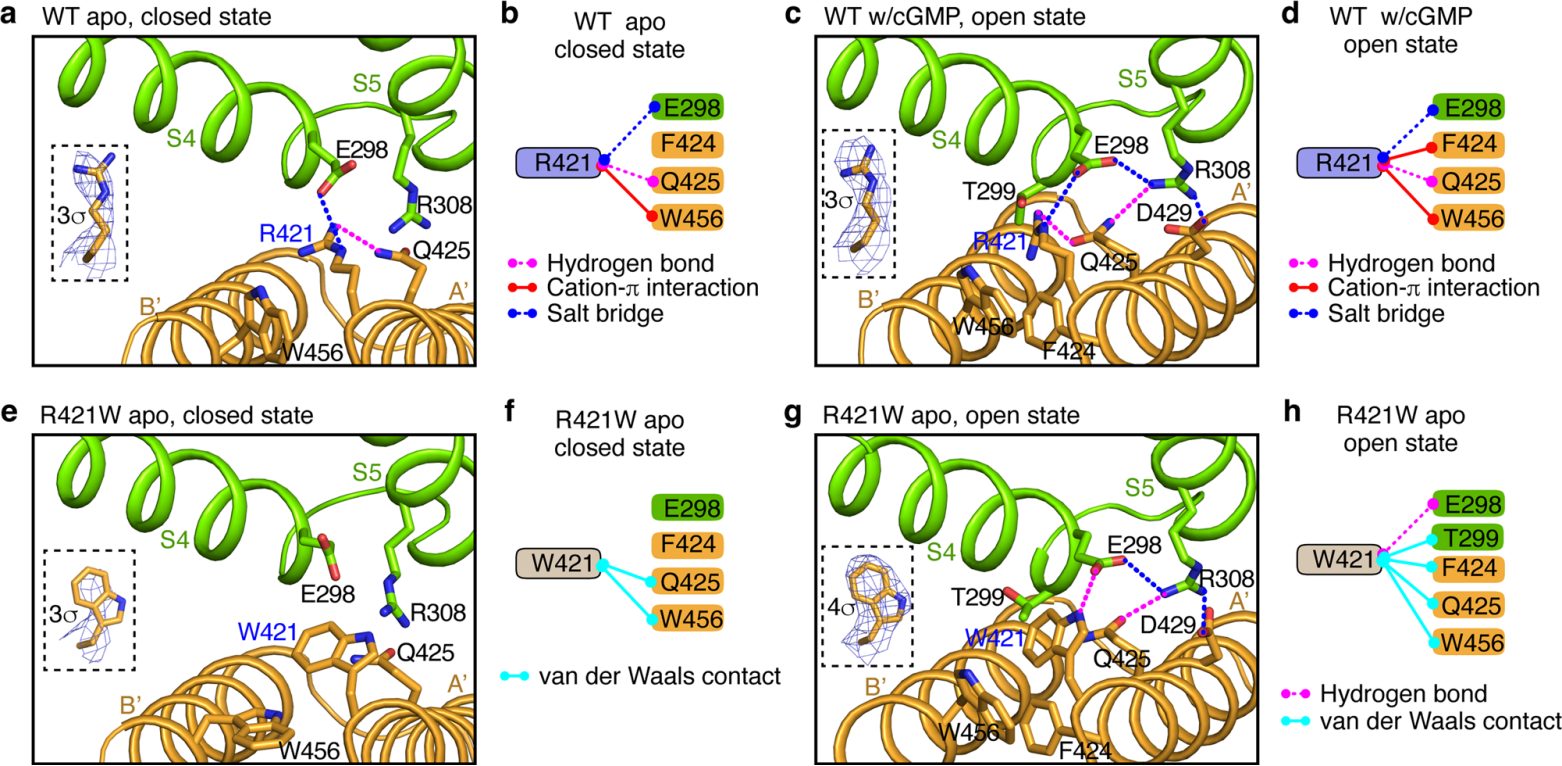
Interaction networks of R421 and W421. **a**, **c**, **e**, **g** Close-up views of local structures and amino acids near R421 in apo closed-state WT TAX-4 (**a**) or cGMP-bound open-state WT TAX-4 (**c**), or near W421 in apo closed-state TAX-4_R421W (**e**) or apo open-state TAX-4_R421W (**g**). Insets show the density maps of R421 or W421 side chains and the corresponding contour levels. **b**, **d**, **f**, **h** Diagram of interactions of R421 or W421 with nearby amino acids in channels and states corresponding to those depicted in **a**, **c**, **e** and **g**, respectively.

How does the R410W mutation cause the channel to open spontaneously? Close inspection of WT and mutant TAX-4 structures provides some answers. R421 is situated in the A’ α helix of the gating ring, which undergoes large cGMP-induced conformational changes in the WT channel^25^. In the closed state, R421 forms a salt bridge with E298 in S4 of a neighboring subunit; it also forms a hydrogen bond with Q425 in the A’ α helix and a cation-π interaction with W456 in the B’ α helix of the gating ring of the same subunit (Fig. 7a, b). The R421-E298 salt bridge is the only interaction between R421 and the transmembrane segments. In the open state, R421 maintains its interactions with E298, Q425 and W456 but also interacts with F424 in A’ (Fig. 7c, d). Furthermore, R421, E298 (in S4), R308 (in S5), Q425 (in A’) and D429 (in A’) form a strong salt bridge/hydrogen bond interaction network (Fig. 7c). Thus, R421 plays a critical role in stabilizing both closed and open states. The R421W mutation alters these interactions. In particular, the closed state is greatly destabilized due to the disruption of the R421-E298 intersubunit salt bridge and the resulting repulsion between the π system of W421 and the negative charge of E298. The intrasubunit R421-Q425 hydrogen bond and R421-W456 cation-π interaction observed in the closed state are also disrupted by the R421W mutation, albeit W421 still makes van der Waals contacts with Q425 and W456 (Fig. 7e, f). In the apo open state, W421 maintains its interactions with E298, F424, Q425 and W456, and recruits T299 in S4 into its van der Waals contacts (Fig. 7g, h). Importantly, the W421-E298-R308-Q425-D429 quintet interaction network remains largely intact (Fig. 7g). These interactions together keep the gating ring and S4, S5 and S6 in the open conformation. These structures confirm the critical role of the gating ring in CNG channel allosteric gating and illustrate how this allosteric gating can be altered by a single mutation. The observation that the open-state structures of apo TAX-4_R421W and cGMP-bound WT TAX-4 are virtually identical, from the mutation site (Fig. 7c, g) to the cavity gate and CNBD (Fig. 3b, d), offers a vivid structural demonstration and manifestation of allostery and microscopic reversibility.

Our finding that an apparent CNG channel LOF DAM opens the channel and produces spontaneous channel activity raises the question of whether more reported LOF missense DAMs also have the same effect, and suggests that a reevaluation of some already characterized missense DAMs and an investigation of yet uncharacterized missense DAMs in rod CNGA1/CNGB1 and cone CNGA3/CNGB3 channels may be necessary, informative and useful. A comprehensive characterization of missense DAMs at the structural and cellular levels may provide not only further mechanistic insights into CNG channel structure, function and channelopathy but also valuable clues for developing therapeutic strategies for patients carrying particular mutations that cause visual disorders.

## Methods

### Molecular biology

Cloning of the *Caenorhabditis elegans* TAX-4 (NCBI Reference Sequence: NP_499033.1) coding sequence was conducted as previously described^26^. Briefly, full-length TAX-4 was cloned into a modified pFastBac1 vector for the expression of MBP-tagged TAX-4 protein and into the pEGFP vector for electrophysiology experiments. The R421W mutation of TAX-4 was generated by using the Q5^®^ Site-Directed Mutagenesis Kit (New England Biolabs) and verified by sequencing.

cDNAs encoding human CNGA3 (NCBI Reference Sequence: NP_001289.1) and CNGB3 (NCBI Reference Sequence: NP_061971.3) were amplified by RT-PCR from HEK 293T cells (ATCC) using SuperScript™ III First-Strand Synthesis System (Thermo Fisher Scientific). Full-length CNGA3 was cloned into the pEZT-BM vector^55^ with an N-terminal MBP tag followed by a P3C cleavage site and a C-terminal FLAG tag for protein expression and purification, or with an N-terminal FLAG tag for whole-cell recording, Ca^2+^ imaging and cell viability assay. Full-length CNGB3 was cloned into the same vector with an N-terminal 2× Strep tag for all studies. The CNGA3 R410W mutation was created using the same method as mentioned above.

### Cell culture

HEK 293T cells (ATCC) were cultured in DMEM (HyClone) supplemented with 10% newborn calf serum (NCS) (Gibco), 100 U ml^−1^ penicillin (Biological Industries), and 0.1 mg ml^−1^ streptomycin (Biological Industries) at 37 °C. In total, 661W cells were obtained from Dr. M. Al-Ubadi at University of Houston with a material transfer agreement and were cultured with the same medium as described above for HEK 293T cells. Sf9 cells (Invitrogen) were cultured in ESF 921 Insect Cell Culture Medium (Expression Systems) at 27 °C. HEK 293 S GnTi^−^ cells (ATCC) were cultured in FreeStyle™ 293 Expression Medium (Thermo Fisher Scientific) supplemented with 2% fetal bovine serum (Sigma-Aldrich) at 37 °C. None of the cell lines was authenticated or tested for mycoplasma contamination.

### Protein expression and purification

Protein expression and purification of full-length WT TAX-4 and TAX-4_R421W were conducted following the procedure reported previously^25^, with several modifications for specific experiments. (1) cGMP was omitted from all purification buffers. (2) For liposome reconstitution, the purified MBP-tagged WT or R421W mutant TAX-4 was incubated overnight with TEV protease (100 μg per 1 mg MBP-tagged protein) and further purified by gel-filtration with a buffer containing 150 mM NaCl, 0.05% LMNG, 5 mM β-mercaptoethanol and 50 mM HEPES, pH adjusted to 8.58 with NaOH. The peak fractions corresponding to WT TAX-4 or TAX-4_R421W tetramers were pooled and concentrated before liposome reconstitution. (3) For cryo-EM study, the purified MBP-tagged WT TAX-4 or TAX-4_R421W was reconstituted into lipid nanodiscs using the same method as published before^25^. After overnight digestion by TEV, the sample was purified by gel-filtration with a buffer containing 500 mM NaCl, 5 mM β-mercaptoethanol and 50 mM HEPES, pH adjusted to 7.4 with NaOH. The eluted peak corresponding to reconstituted WT TAX-4/nanodisc or TAX-4_R421W/nanodisc complex was collected and concentrated for cryo-EM analysis. (4) cGMP-bound TAX-4_R421W was prepared by incubation with 2 mM cGMP for 2 h before grid freezing. (5) Apo WT TAX-4 was incubated with 5 mM EGTA for 2 h before grid freezing.

WT and R410W mutant human CNGA3/CNGB3 heterotetramers were expressed in HEK 293S GnTi^−^ cells using the BacMam system. Recombinant baculoviruses of full-length WT and R410W mutant CNGA3 and WT CNGB3 were generated separately with Sf9 insect cells (Invitrogen) following the standard protocol. Harvested baculoviruses were amplified twice in Sf9 cells to obtain sufficient viruses for large-scale infection. HEK 293 S GnTi^−^ cells were grown in suspension at 37 °C. When cells reached a density of 2–2.5 × 10^6^ cells ml^−1^, a baculovirus mixture of WT or R410W mutant CNGA3 and WT CNGB3 at a ratio of 1:1.5 was added to the culture (10%, v/v). After incubation for 12–24 h, the culture was supplemented with 10 mM sodium butyrate to boost the expression, and was further incubated at 30 °C for 72 h before collection.

Purification of WT and R410W mutant CNGA3/CNGB3 heterotetramers was carried out at 4 °C. Cell pellet from 4 l culture was resuspended and lysed by stirring for 30 min in 150 ml hypotonic buffer (10 mM HEPES-Na, pH 8.58, 1 mM TCEP, 2 mM EGTA) supplemented with a protease inhibitor cocktail (Sigma). Membrane fraction was collected by centrifugation at 29,448 × *g* for 40 min, and then homogenized with a Dounce homogenizer in 150 ml extraction buffer (50 mM HEPES-Na, pH 8.58, 150 mM NaCl, 2% DDM, 0.2% CHS, 1 mM TCEP, 2 mM EGTA). After incubation for 1 h, the solubilized membrane was clarified by ultracentrifugation at 111,338 × *g* for 50 min. The supernatant was incubated with amylose resin (New England Biolabs) for 2 h with gentle agitation. The resin was collected by low-speed spin at 800 × *g*, transferred into a gravity column, and washed with 50 column volume (CV) of wash buffer (WB) (50 mM HEPES-Na, pH 8.58, 150 mM NaCl, 0.1% DDM, 0.01% CHS, 1 mM TCEP, 2 mM EGTA). The protein was eluted from amylose resin with 30 ml WB containing 20 mM maltose, and then loaded onto a 1 ml StrepTrap HP column (Cytiva). The column was washed with 20 CV of WB and eluted with 10 ml WB containing 5 mM d-Desthiobiotin. Eluted protein was incubated with P3C protease overnight to cleave the MBP tag and was further purified by a Superose 6 column (Cytiva) equilibrated with a gel-filtration buffer (50 mM HEPES-Na, pH 8.58, 150 mM NaCl, 0.1% DDM, 0.01% CHS, 1 mM TCEP, 1 mM EGTA).

### Cryo-EM sample preparation and data acquisition

Cryo-EM grids were prepared by applying 3 μl of TAX-4_R421W/nanodisc sample (1.2 mg ml^−1^ with or without 2 mM cGMP) or 3 μl of WT TAX-4/nanodisc sample (1.2 mg ml^−1^ without cGMP) to a glow-discharged C-flat™ CF-1.2/1.3-3Au holey carbon grid (Protochips), which was manually prefabricated with gold supports^56^. After waiting for 10 s, the grid was blotted for 8 s (double-sided, blot force 3) under 100% humidity and 4 °C using FEI Vitrobot Mark IV (FEI) and immediately plunged into liquid ethane cooled by liquid nitrogen. For cGMP-bound and apo TAX-4_R421W samples, micrographs were acquired by a Titan Krios microscope (Thermo Fisher) operated at 300 kV and equipped with a K3 direct electron detector (Gatan) working at counting mode. Leginon^57^ was used for data collection. A nominal magnification of ×105,000 was used for imaging the samples, corresponding to a final pixel size of 0.83 Å on image. The defocus ranged from −0.9 to −1.5 μm. For the cGMP-bound TAX-4_R421W sample, a total of 4582 movies were collected. Each micrograph was dose-fractionated to 60 movie frames under a dose rate of 16 counts per pixel per second, with a total exposure time of 3 s and a frame exposure time of 50 ms, resulting in a total dose of 69.7 e^−^/Å^2^. For the apo TAX-4_R421W sample, a total of 4632 movies were collected. Each micrograph was dose-fractionated to 50 movie frames under a dose rate of 16 counts per pixel per second, with a total exposure time of 2.5 s and a frame exposure time of 50 ms, resulting in a total dose of 58 e^−^/Å^2^. For the apo WT TAX-4 sample, micrographs were collected on a Titan Krios microscope (Thermo Fisher) operated at 300 kV and equipped with a K2 direct electron detector (Gatan) working at counting mode. A nominal magnification of ×22,500 was used for imaging the samples, corresponding to a final pixel size of 1.073 Å on image. The defocus ranged from −0.9 to −3.0 μm. A total of 2494 movies were collected using Leginon^57^. Each micrograph was does-fractionated to 50 movie frames under a dose rate of 8 counts per pixel per second, with a total exposure time of 10 s and a frame exposure time of 200 ms, resulting in a total dose of 69.80 e^−^/Å^2^.

### Image processing

For all cryo-EM data collected, drift correction, beam-induced motion correction and dose-weighting were performed with MotionCor2^58^ using a 5 × 5 patch. The resulting integrated micrographs were used for further processing. Contrast-transfer function parameters of the micrographs were estimated using Gctf^59^. Particles were automatically picked by RELION 3.0^60^. For cGMP-bound and apo TAX-4_R421W samples, 3,988,515 and 1,679,420 particles were initially picked, respectively. The picked particles were 4 × binned (3.32 Å) and applied to two rounds of 2D classification and one round of 3D classification with *C*1 symmetry. After 2D and 3D classification, 284,615 and 193,718 particles were selected respectively for cGMP-bound and apo samples, according to the classes exhibiting recognizable TAX-4 channel features. For the cGMP-bound sample, the selected particles were subjected to CryoSPARC^61^ for 3D refinement with *C*4 and *C*1 symmetry individually. The final 3D cryo-EM density map of cGMP-bound TAX-4_R421W was reconstructed and refined at a 2.92 Å resolution with *C*4 symmetry and a 2.93 Å resolution with *C*1 symmetry. For the apo sample, the selected particles were used to run a consensus 3D refinement in RELION. The resulting major class was initially subjected to a standard 3D classification with a simple solvent mask. This produced a density map that displayed obvious heterogeneity (Supplementary Fig. 2e). Notably, the densities of the gate residues (F403 and V407), the C-linker, and the C helix of the CNBD could be fitted with both closed and open structures of WT (Supplementary Fig. 2f, g), suggesting that this map was a mixture of both open and closed states. Thus, a mask containing S6, the gating ring and the CNBD was created, because these regions show the largest conformational changes during cGMP activation^25^. A focused 3D classification (*K* = 8) was performed by applying this mask. After focused 3D classification, the largest class and the second largest class exhibited two different conformations. The corresponding particles in those two classes were separately passed to CryoSPARC for 3D refinement. Finally, the density map of the open state of apo TAX-4_R421W was refined at a 3.05 Å and a 3.21 Å resolution with *C*4 and *C*1 symmetry, respectively. The density map of the closed state of apo TAX-4_R421W was refined at a 3.16 Å and a 3.40 Å resolution with *C*4 and *C*1 symmetry, respectively. For the apo WT TAX-4 sample, 1,027,231 particles were initially picked, 4 × binned (4.29 Å), and applied to two rounds of 2D classification and one round of 3D classification with *C*1 symmetry. After 2D and 3D classification, 210,232 particles were selected and re-extracted without binning (1.073 Å), and then used to run a consensus 3D refinement in RELION. A focused 3D classification (*K* = 8) was performed by applying the same mask including S6, the gating ring and the CNBD as that used for the apo TAX-4_R421W sample (Supplementary Fig. 2c). After focused 3D classification, particles from the largest two classes possessing 63.6 and 21.2% of the particles were individually selected and passed to CryoSPARC for 3D refinement. After 3D refinement, both classes showed an apo closed state that is identical to the one we published before^25^.

### Model building, refinement, and validation

The previously solved open-state (PDB ID: 6WEK) and closed-state (PDB ID: 6WEJ) structures of WT TAX-4 in nanodiscs were used respectively as a reference model for model building of the open states of cGMP-bound and apo TAX-4_R421W and the closed state of apo TAX-4_R421W. The reference model was docked onto the corresponding cryo-EM map constructed with *C*4 symmetry using UCSF Chimera^62^. Afterward, model building was carried out in Coot^63^ using the tool of Real-Space Refinement Zone by manually adjusting main chain/side chain conformations. R421 was mutated to tryptophan with the Mutate & AutoFit tool. After building an initial model, coordinates and individual B-factors were refined against summed maps with secondary structure restraints and strict NCS constraints using phenix.real_space_refine^64^ implemented in PHENIX^65^. Overfitting of the atomic models was validated via previously described methods^66^. In brief, atoms in the final model were randomly shifted by up to 0.2 Å, and the new model was then refined against one of the two half maps generated during the final 3D reconstruction. FSC values were calculated between the map generated from the resulting model and the two half maps, as well as the averaged map of the two half maps. The quality of the models was evaluated with MolProbity^67^. All the figures were prepared in PyMol^68^ or UCSF Chimera^62^.

### Electrophysiology

Whole-cell recordings were performed on HEK 293T cells transfected with WT or mutant TAX-4 or CNGA3/CNGB3. LipoD293™ (SignaGen Laboratories) was used in all transfections. For TAX-4, cells were transfected with GFP-tagged TAX-4 or TAX-4_R421W plasmids. For human CNGA3/CNGB3, cells were transfected with a mixture of enhanced GFP, CNGB3, and CNGA3 or CNGA3_R410W plasmids in a 1:5:1 ratio. Cells were used for recording 48 h after transfection. All experiments were performed at room temperature (22–23 °C). Pipettes were fabricated from borosilicate glass (Corning Pyrex) using a micropipette puller (PC-10; Narishige) and were fire-polished to resistances of 2−4 MΩ for whole-cell recording. Whole-cell currents were elicited by 30-ms voltage steps from −100 to +100 mV with 10-mV increments, with a holding potential of 0 mV. Currents were amplified by Axopatch 200B and digitized by Digidata 1322A (Molecular Devices). Currents were low-pass filtered at 1 kHz and sampled at 10 kHz. pCLAMP 8.2 software (Molecular Devices) was used for data acquisition and analysis. Both intracellular and extracellular solutions contained 140 mM NaCl, 5 mM KCl, 1 mM EGTA and 10 mM HEPES (pH 7.4 adjusted with NaOH). The EC_50_ of cGMP activation is 0.16–0.34 μM for TAX-4^26,69^ and 9.8–18 μM for CNGA3 homomeric channels^3,70^. To ensure full activation of WT and mutant channels, a high concentration of 100 μM cGMP was added to the pipette solution.

For inside-out patch-clamp recordings on giant unilamellar vesicles (GUV) (i.e., liposomes), purified WT or mutant channel proteins were reconstituted at a 1:100 (w/w) ratio into preformed Triton X-100 (0.11%, w/v)-destabilized liposomes that were prepared from Brain Extract Polar (Avanti Polar Lipid) in 150 mM NaCl and 20 mM HEPES (pH 7.5 adjusted with NaOH) as described^71^. Triton X-100 was subsequently removed by Bio-Beads SM-2 (BioRad). In total, 3 µl of reconstituted proteoliposome was dehydrated on a clean cover slip overnight at 4 °C. The dried proteoliposome layer was then rehydrated at room temperature with a bath solution containing 270 mM NaCl, 30 mM KCl and 10 mM HEPES 10 (pH 7.5 adjusted with NaOH) to form GUV. Single-channel recording pipettes were pulled from borosilicate glass (Corning Pyrex) and had a resistance of 5–7 MΩ when filled with the bath solution. Giga-Ω seal was formed by gentle suction when the patch pipette made contact with the GUV. To obtain an inside-out single-layer of membrane patch, the pipette was pulled away from the GUV, and the tip was exposed to air for 1–2 s and put back into the bath solution. To elicit single-channel currents, 5–10 µM cGMP was added to the bath solution. Data were acquired and analyzed as described above for whole-cell recordings.

Single-channel current amplitude histograms were generated and fitted with the Gaussian distribution using Prism 7, and single-channel openings were determined using a 50% threshold of the full amplitude. Because most of the recordings lasted only a few minutes, the currents were mostly recorded with only one concentration of cGMP (5 μM or 10 μM) and at only one or a few voltages, typically −100 mV, −80 mV, +80 mV or +100 mV. Previous recordings of heterologously expressed CNGA3/CNGB3 channels show that their single-channels have a largely linear current-voltage relationship between −100 mV and +100 mV, with a slight inward or outward rectification between +70 and +100 mV^21,45^; thus for analyzing single-channel conductance and open probability (P_o_), we pooled the results at different voltages together, with one voltage for each patch. Some patches were not included in the P_o_ analysis because of noisy baseline. Data were presented as mean ± SD.

### Intracellular Ca^2+^ imaging

HEK 293T and 661W cells were transfected with a mixture of DsRed (for HEK 293T cells) or mCherry (for 661W cells), CNGB3, and CNGA3 or CNGA3_R410W plasmids in a 1:5:1 ratio, and were cultured in the same way as described above. Four and six separate cultures of 661W and HEK 293T cells, respectively, were made. Intracellular calcium imaging of transfected cells was performed using a LSM 800 (Zeiss) confocal microscope. For each culture, cells transfected with each channel type were plated in 4 wells of a 24-well plate and, 48 h after transfection, were loaded with 10 µM Fluo-4 AM (Invitrogen) and 0.02% Pluronic F-127 (Molecular Probes) at 37 °C for 1 h in a Ca^2+^-free imaging solution (145 mM NaCl, 5 mM KCl, 1 mM MgCl_2_, 10 mM HEPES, pH 7.4 adjusted with NaOH). The cells were alternately exposed to 561 nm and 488 nm lasers for 1.27 s to excite mChery/DsRed and Fluo-4 AM, respectively, every 20–30 s for 7 min after being treated with each of the following conditions:

1. Imaging solution without Ca^2+^.
2. Imaging solution with 2 mM CaCl_2_ and 100 µM of L-*cis*-diltiazem (DTZ).
3. Washout with Ca^2+^-free imaging solution.
4. Imaging solution with 2 mM CaCl_2_.
5. Imaging solution with 2 mM CaCl_2_ and 100 µM CPT-cGMP.

Seven minutes of imaging were chosen because in all the conditions the fluorescence change peaked within this time period.

Because of limited microscope time, usually 2 or 3 wells were imaged for each channel type in each culture and not all wells transfected with CNGA3_R410W/CNGB3 were treated with CPT-cGMP. In some experiments, cells in some wells detached from the plates because of the solution exchanges, floated around and often stayed in the field of view, and thus distorted imaging. These wells were discarded. This occurred to both cell types and more often with 661W cells. As such, images from only of 5–6 wells of 661W cells and 10–11 wells of HEK 293T cells were of sufficient quality for analysis.

Relative fluorescence unit (RFU) of red (from mCherry or DsRed) and green (from Fluo-4 AM) was measured using ZEISS ZEN 3.2 software. For each well of cells, the image with the highest green RFU was selected for analysis, based on a plot of green RFU over the 7-min imaging period (Supplementary Fig. 10). For each image, the green RFU was normalized using the accompanying red RFU, which served as an indicator for the overall transfection efficiency. More importantly, this normalization reduces the effect of photobleaching and defocusing/refocusing, which took place sometimes after a solution exchange.

Relative signal increase (RSI) of each well caused by spontaneous channel activities was calculated using the following formula:

1 $${{{{{{\rm{RSI}}}}}}}_{{{{{{\rm{sp}}}}}}}=\frac{{{{{{{\rm{RFU}}}}}}}_{{{{{{{\rm{Ca}}}}}}}^{2+}}-{{{{{{\rm{RFU}}}}}}}_{0{{{{{{\rm{Ca}}}}}}}^{2+}}}{{{{{{{\rm{RFU}}}}}}}_{{{{{{{\rm{Ca}}}}}}}^{2+}{{\& \; DTZ}}}-{{{{{{\rm{RFU}}}}}}}_{0{{{{{{\rm{Ca}}}}}}}^{2+}}}\,-1$$

One well with ${{{{{{\rm{RFU}}}}}}}_{{0{{{{{\rm{Ca}}}}}}}^{2+}}$ > ${{{{{{\rm{RFU}}}}}}}_{{{{{{{\rm{Ca}}}}}}}^{2+}}$ was discarded in data analysis.

RSI of each well caused by the addition of 100 µM CPT-cGMP was calculated using the following formula:

2 $${{{{{{\rm{RSI}}}}}}}_{{{{{{\rm{cGMP}}}}}}}=\frac{{{{{{{\rm{RFU}}}}}}}_{{{{{{{\rm{cGMP\& Ca}}}}}}}^{2+}}-{{{{{{\rm{RFU}}}}}}}_{0{{{{{{\rm{Ca}}}}}}}^{2+}}}{{{{{{{\rm{RFU}}}}}}}_{{{{{{{\rm{Ca}}}}}}}^{2+}}-{{{{{{\rm{RFU}}}}}}}_{0{{{{{{\rm{Ca}}}}}}}^{2+}}}\,-1$$

### Surface protein biotinylation

Pierce^TM^ Cell Surface Biotinylation and Isolation Kit (Thermo A44390) was used for HEK 293T surface biotinylation. Briefly, HEK 293T cells were split into 6-cm dishes and transfected with WT CNGA3 or CNGA3_R410W and CNGB3 as described above. All procedures were performed at 4 °C. In total, 48 h after transfection, each dish was biotinylated in PBS containing 1 mg/ml sulfo-NHS-SS-biotin for 1 h. TBS (25 mM Tris, 150 mM NaCl, pH 7.2) was added for quenching for 5 min. Each dish was washed 2 additional times with ice-cold TBS. Cells were harvested from the dish using a cell scraper and was washed twice with TBS and lysed with Pierce IP lysis buffer (Thermo 87787) supplemented with a protease inhibitor mixture for 1 h with vortexing every 5 min. The homogenate was centrifuged at 10,000 × *g* for 10 min and the supernatant was incubated with neutravidin-agarose beads overnight with rotation. The next morning, flow-through was collected by brief spin (1000 g, 1 min) and saved for Western blot (this is the flow-through fraction). The beads were washed 4 times with a WB containing a mixture of protease inhibitors. Finally, the beads were incubated with SDS sample buffer containing β-mercaptoethanol at room temperature (22–23 °C) for 30 min with rotation to elute the surface biotinylated proteins (this is the elution fraction).

### Western blot

For SDS-PAGE, samples were run in home-made 4–12% acrylamide gels. Electrical transfer to PVDF membranes (BioRad) was performed in a standard 20 mM Tris, 192 mM glycine, pH~8.3 buffer for 70 min at 100 V, on ice. Membranes were handled in PBS containing 0.2% Tween-20 (PBST). Blocking buffer was 10% NCS in PBST. Membranes were blocked for 2–3 h at room temperature and incubated with primary antibodies with blocking buffer overnight at 4 °C with gentle orbital shaking. Membranes were washed 3–4 times with PBST before incubated with secondary antibodies diluted with blocking buffer. Primary antibodies were used at 1:1000–1:2000 dilutions and secondary antibodies at 1:2000 dilutions. Protein bands were visualized using enhanced chemiluminescence reagents on X-ray film. Anti-FLAG antibody was purchased from Sigma (A2220). Anti-Strep antibody was purchased from Sigma (71591-3). Anti-actin antibody was purchased from Sigma (A2066). All secondary antibodies were purchased from Santa Cruz biotechnology. Actin was used as a loading control and as an indicator of the healthiness and integrity of the cells used for surface biotinyation. A lack of actin in the elution fraction indicates healthy cells.

### Cell viability assay

HEK 293T and 661W cells were transfected with CNGB3 and CNGA3 or CNGA3_R410W plasmids in a 5:1 ratio using FuGene HD reagent (Promega). Cells were cultured in the same way as described above and were split into 96-well clear-bottom tissue culture plates 6 h after transfection at a density of ~1 × 10^5^ and 4 × 10^4^ cells per well, respectively. In three cultures, some wells of HEK 293T cells were treated with 10 μM DTZ after the split. Because long-term treatment of DTZ was toxic to HEK 293T cells (Supplementary Fig. 11), and because 661W cells were much more fragile than HEK 293T cells, we did not treat 661W cells with DTZ.

In total, 48 h after transfection, HEK 293T and 661W cells were incubated with Resazurin Assay Kit (Abcam) for 4 h at 37 °C. The fluorescence level was measured at Ex/Em of 530/590 nm according to manufacturer’s protocol and was normalized by the WT mean value.

### Statistics and reproducibility

In Ca^2+^ imaging and cell viability experiments, the statistical significance of the differences between WT and mutant groups was evaluated by using unpaired Student’s *t* test. Data are presented as mean ± standard deviation of the mean. A *p* value of < 0.05 is considered statistically significant. The electrophysiological experiments, intracellular Ca^2+^ imaging and cell viability assays were readily and reliably reproduced. The repeat numbers are described in figure legends.

### Reporting summary

Further information on research design is available in the Nature Research Reporting Summary linked to this article.

## Supplementary information

Supplementary information

Supplementary Data 1

Supplementary Data 2

Reporting Summary

## Data Availability

The data supporting the findings of this study are available within the paper. The cryo-EM density maps for cGMP-bound open state, apo open state, and apo closed state of TAX-4_R421W have been deposited to the Electron Microscopy Data Bank under accession codes EMD-24113, EMD-24115 and EMD-24114, respectively. The corresponding atomic coordinates have been deposited to the Protein Data Bank under accession codes 7N15, 7N17 and 7N16, respectively. Uncropped western blot and SDS-PAGE gels are provided in Supplementary Data 1. Source data for plotting and statistics are provided in Supplementary Data 2. All other data including cryo-EM density maps in Supplementary Fig. 6 are available from the corresponding author on reasonable request.
