# Supplementary material for: Structural and functional characterization of an achromatopsia-associated mutation in a phototransduction channel
Source: Commun Biol. 2022 Mar 1;5:190. doi: 10.1038/s42003-022-03120-6 (PMC8888761; doi:10.1038/s42003-022-03120-6)
Supplement: Supplementary file 1 — Supplementary information [file 42003_2022_3120_MOESM1_ESM.pdf]

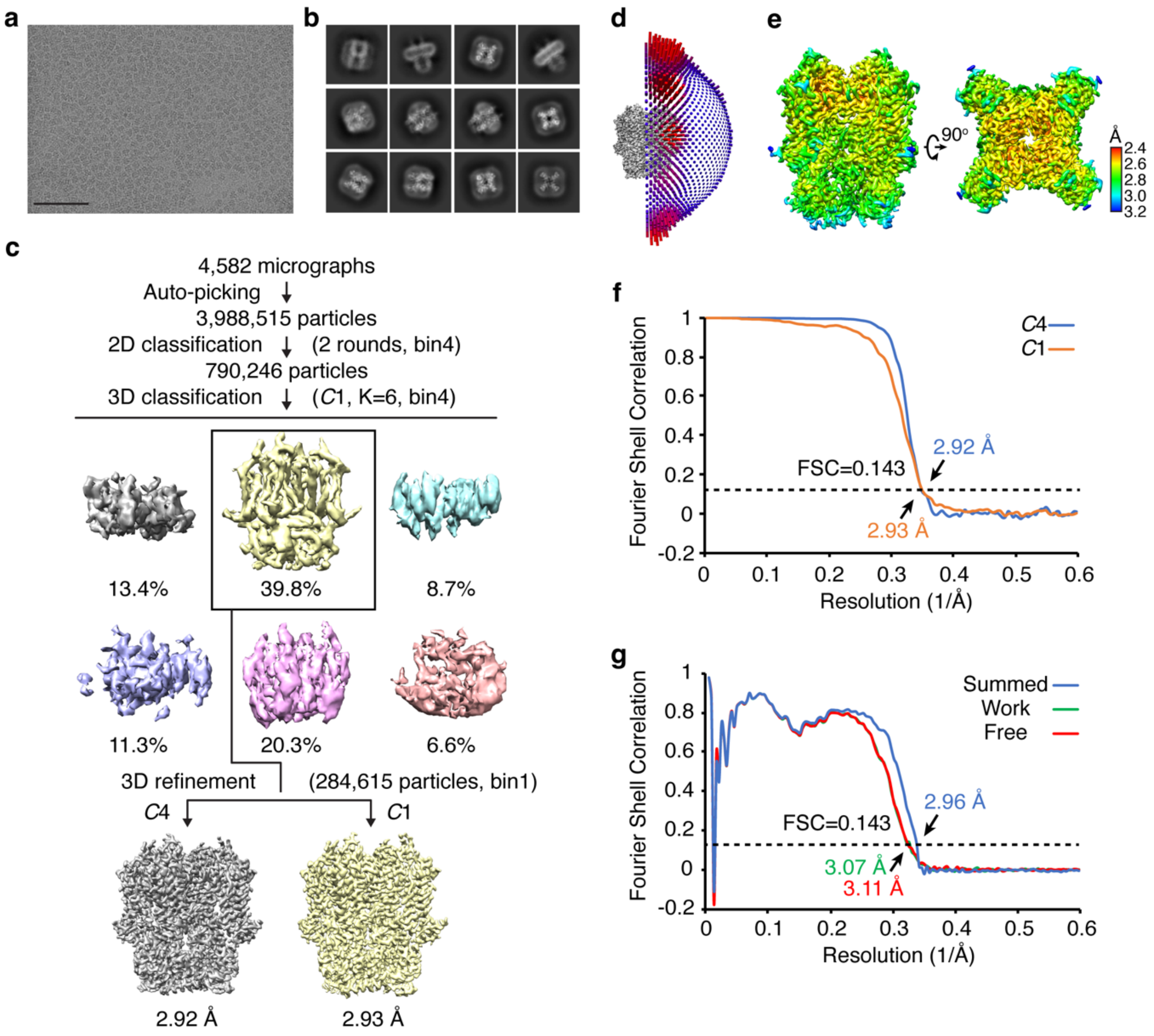

Supplementary Fig. 1 Cryo-EM single-particle analysis of cGMP-bound TAX-4_R421W. a A representative motion-corrected micrograph. Scale bar: 100 nm. **b** Gallery of typical averages from 2D classification. **c** Flow chart of cryo-EM image processing. **d** Euler angle distribution of particles used in the final 3D refinement with *C*4 symmetry. **e** Local resolution of the final density map refined with *C*4 symmetry. **f** Gold-standard FSC curves of the final 3D refinement with *C*4 and *C*1 symmetry, respectively. **g** FSC curves for cross-validation between maps and model. Blue, model versus the summed map. Green, model versus the half map that was used for model refinement (called ‘work’). Red, model versus another half map that was not used for model refinement (called ‘free’).

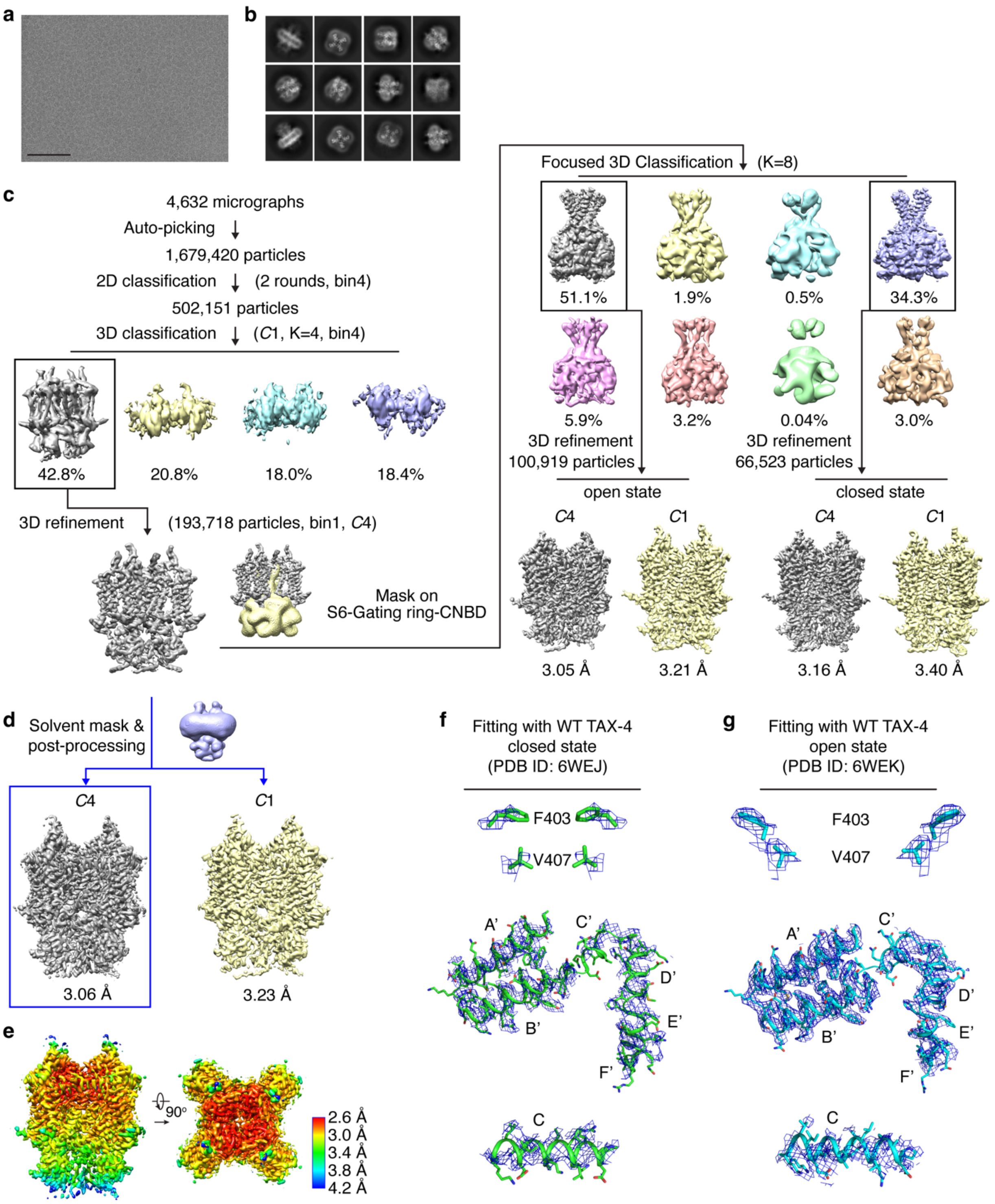

Supplementary Fig. 2 Cryo-EM single-particle analysis of apo TAX-4_R421W. a A representative motion-corrected micrograph. Scale bar: 100 nm. **b** Gallery of typical averages from 2D classification. **c** Flow chart of cryo-EM image processing. **d**

Consensus 3D refinement using particles from the major group (42.8%) obtained after standard 3D classification. The C4 map in blue square was used for further analysis in **e** to **g**. **e** Local resolution of the consensus 3D map. **f,g** Fitting of the gate-forming residues, the C-linker and the C helix of the CNBD from the WT TAX-4 apo closed state (**f**) and cGMP-bound open state (**g**) into the consensus 3D map. Contour level: 2 σ. More details of **d-g** are described in Image Processing in Methods.

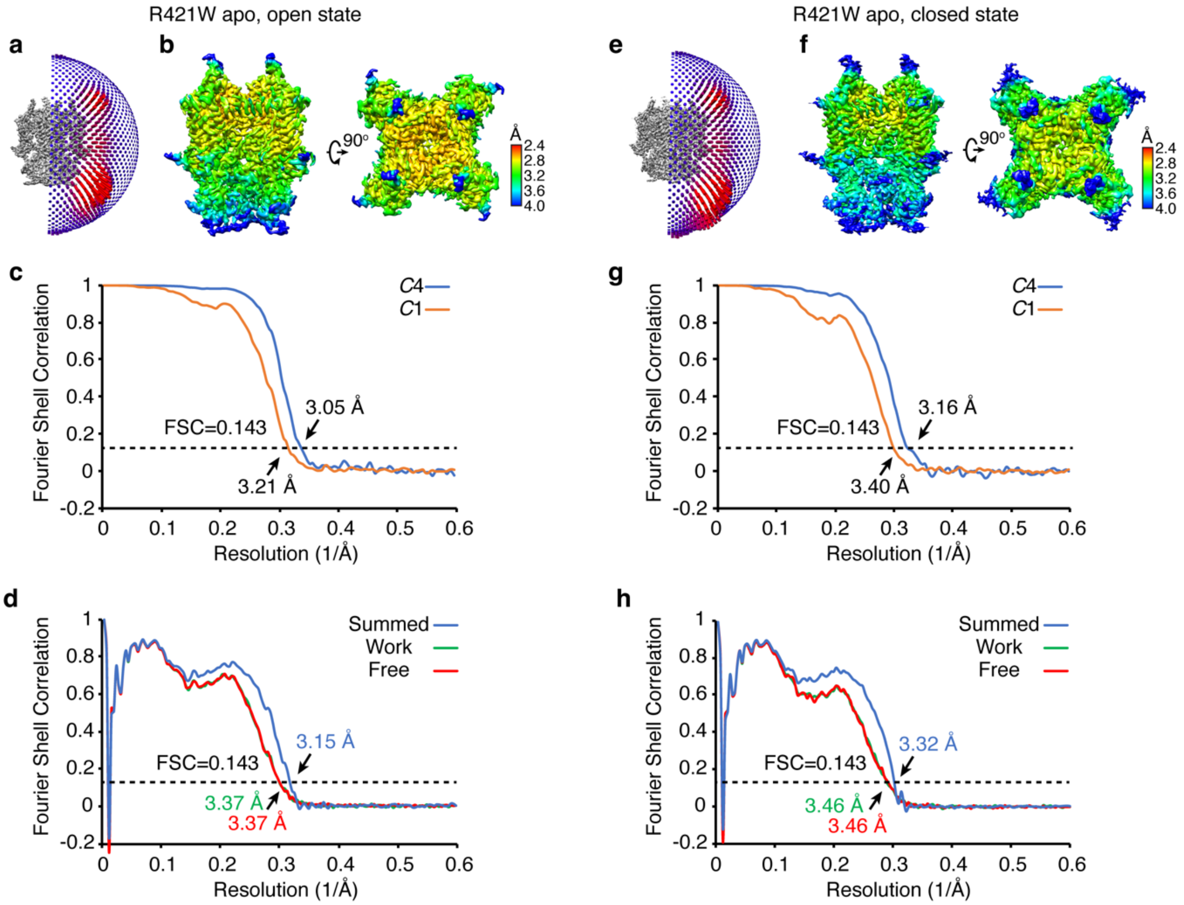

Supplementary Fig. 3 Validation of the open and closed states of apo TAX-4_R421W. a, e Euler angle distribution of particles used in the final 3D refinement with *C*4 symmetry. **b, f** Local resolution of the final density map refined with *C*4 symmetry. **c, g** Gold-standard FSC curves of the final 3D refinement with *C*4 and *C*1 symmetry, respectively. **d, h** FSC curves for cross-validation between maps and model. Blue, model versus the summed map. Green, model versus the half map that was used for model refinement (called 'work'). Red, model versus another half map that was not used for model refinement (called 'free').

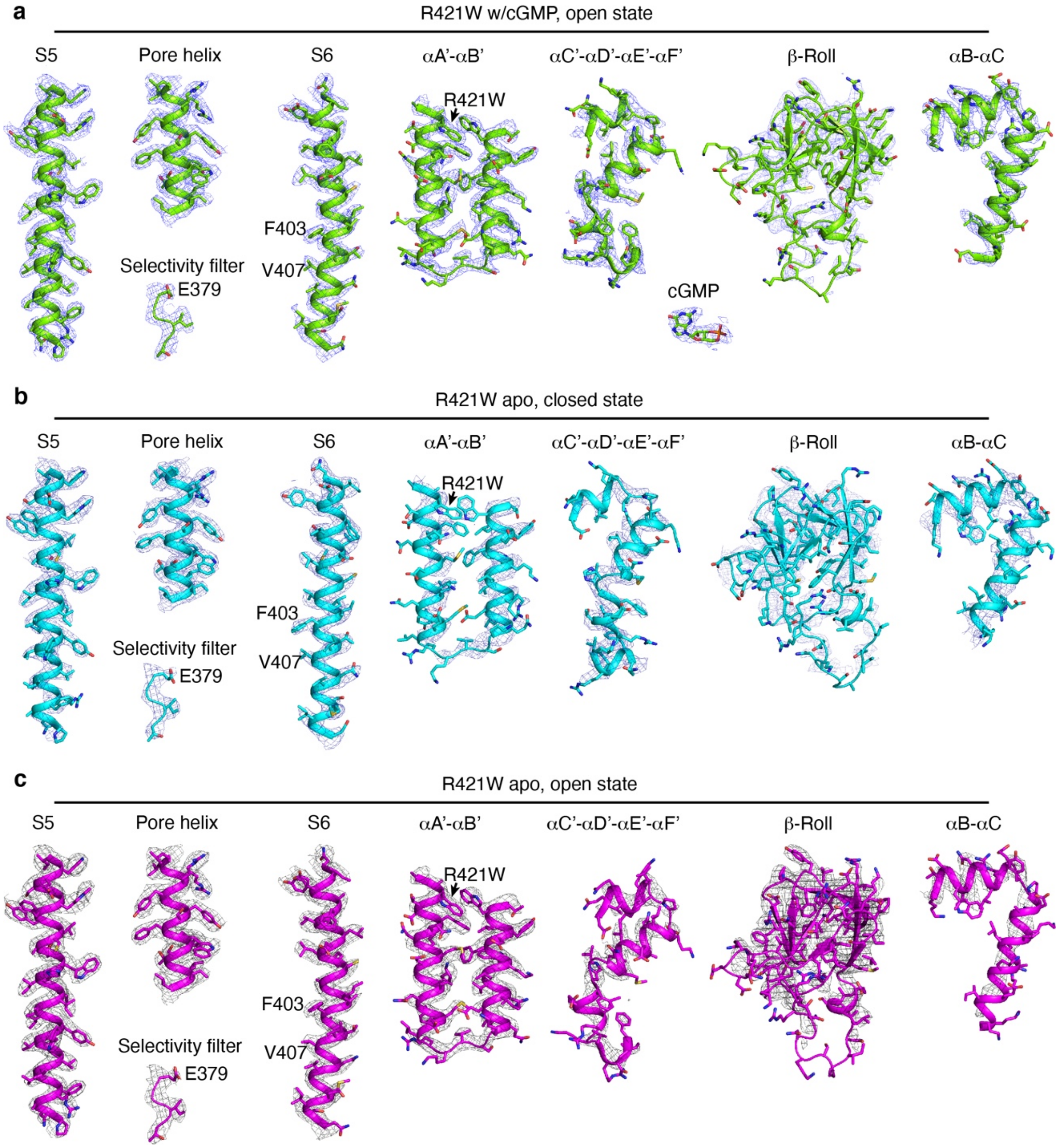

Supplementary Fig. 4 Cryo-EM density maps and atomic models of selected key regions of TAX-4_R421W. a cGMP-bound open state. The map was low-pass filtered to 2.92 Å, sharpened with a temperature factor of –143 Å^2 and contoured at 4σ. **b** Apo closed state. The map was low-pass filtered to 3.16 Å, sharpened with a temperature factor of –116 Å^2 and contoured at 4σ. **c** Apo open state. The map was low-pass filtered to 3.05 Å, sharpened with a temperature factor of –127 Å^2 and contoured at 4σ.

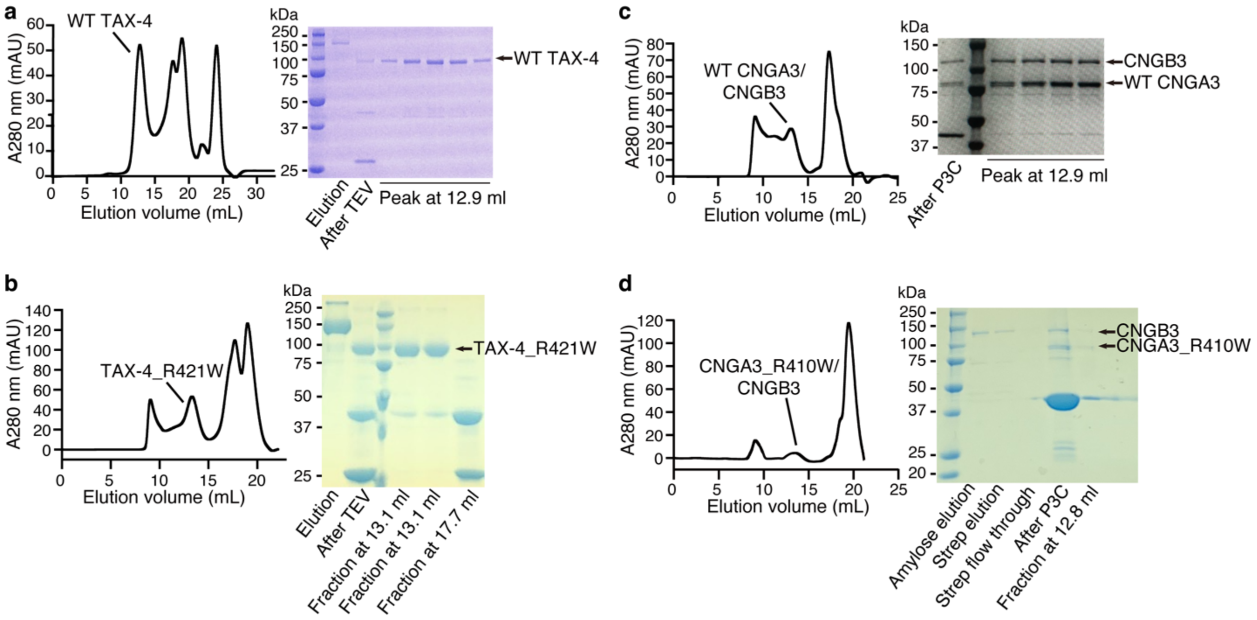

Supplementary Fig. 5 Biochemical characterization of WT and mutant channel proteins. Left panel: Gel filtration profiles of the indicated channel proteins from a superose 6 10/300 GL column. Right panel: Coomassie blue SDS-PAGE gels showing proteins at different purification steps. Arrows indicate target proteins.

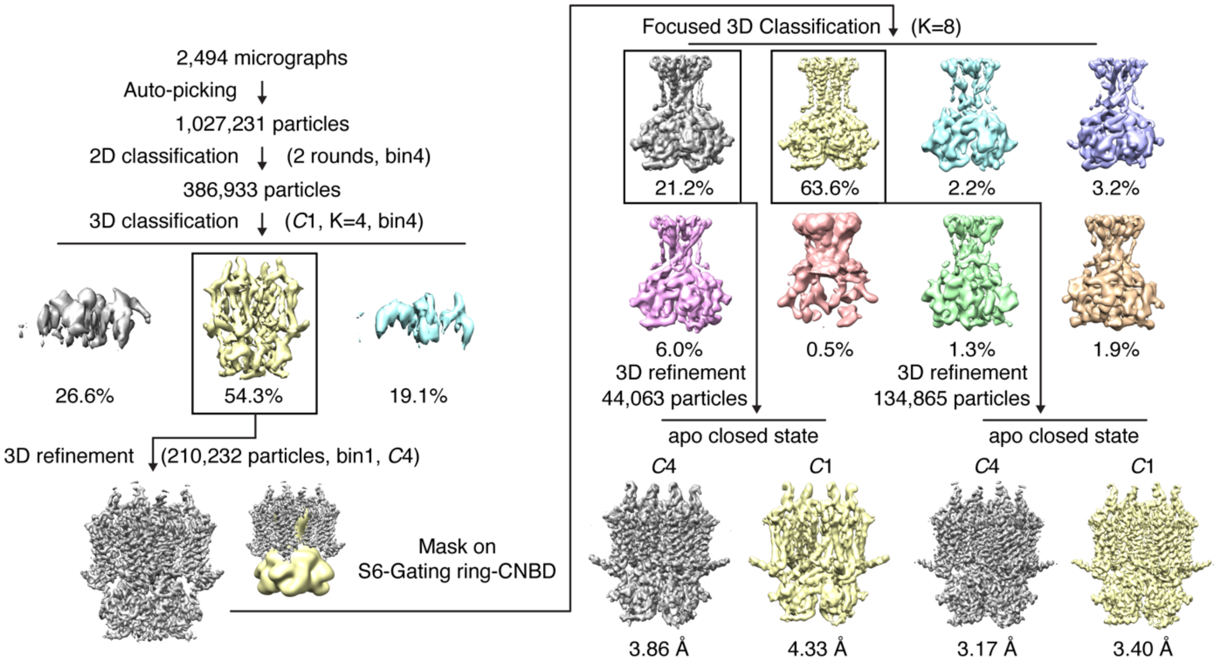

Supplementary Fig. 6 Cryo-EM single-particle analysis of apo WT TAX-4. Flow chart of cryo-EM image processing of apo WT TAX-4 using the same procedures and local mask as those in Supplementary Fig. 2c.

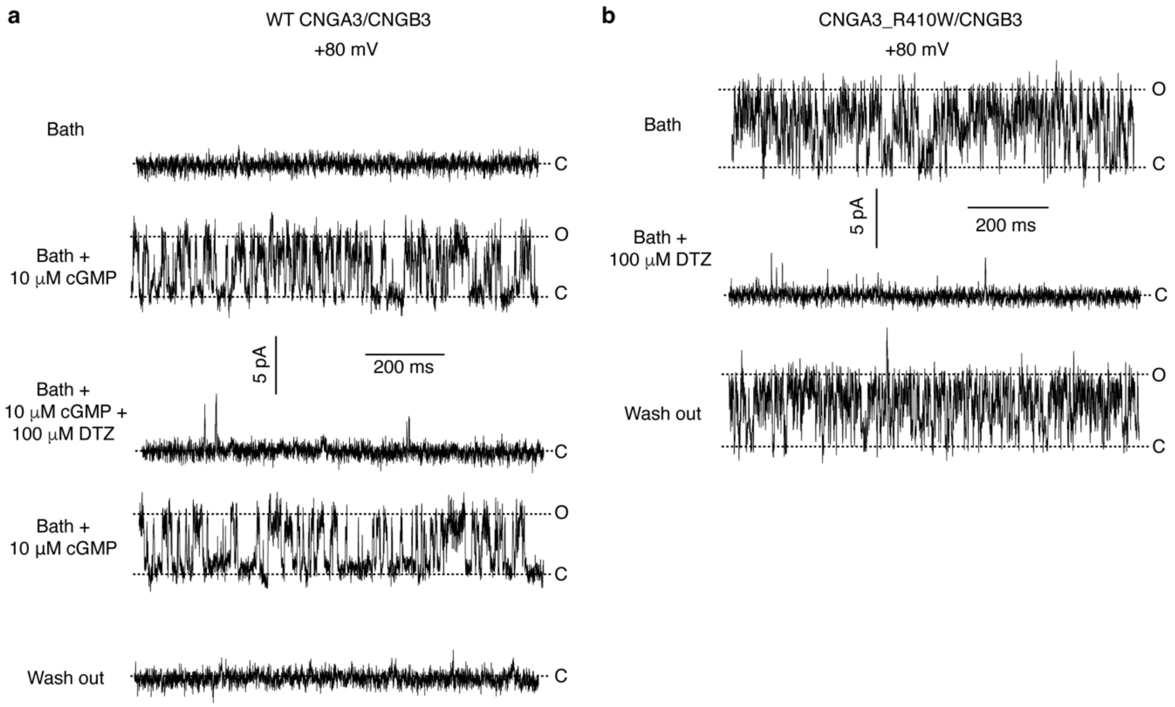

Supplementary Fig. 7 WT and mutant CNGA3/CNGB3 channels are blocked by diltiazem. cGMP-induced single-channel currents in WT CNGA3/CNGB3 (**a**) and spontaneous single-channel currents in mutant CNGA3/CNGB3 (**b**) are reversibly inhibited by 100 µM L-*cis*-Diltiazem (DTZ). Inhibition was also observed in 4 and 3 other patches for WT and mutant channels, respectively.

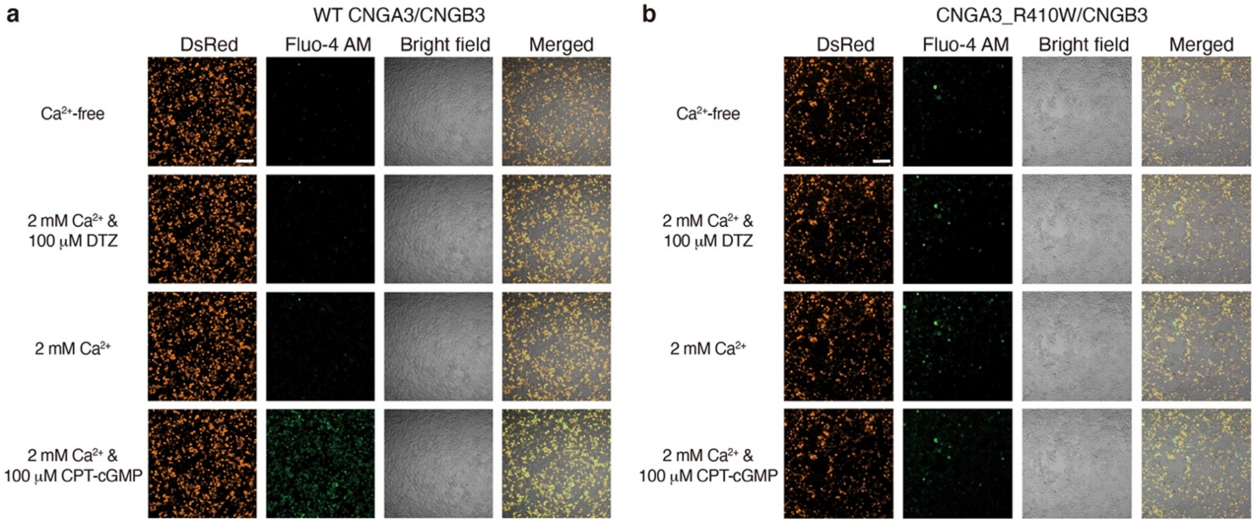

Supplementary Fig. 8 Ca^{2+} imaging of HEK 293T cells transfected with WT or mutant CNGA3/CNGB3. Representative images of HEK 293T cells transfected with WT (**a**) or mutant (**b**) CNGA3/CNGB3. DsRed was used as a normalization factor for cell number and transfection efficiency. Merged images show that most of the cells did not display DsRed signal and/or did not turn green upon the addition of 100 μM CPT-cGMP, indicating a low transfection efficiency. The images were selected from the view fields that had the highest numbers of transfected cells. Scale bars: 100 μm.

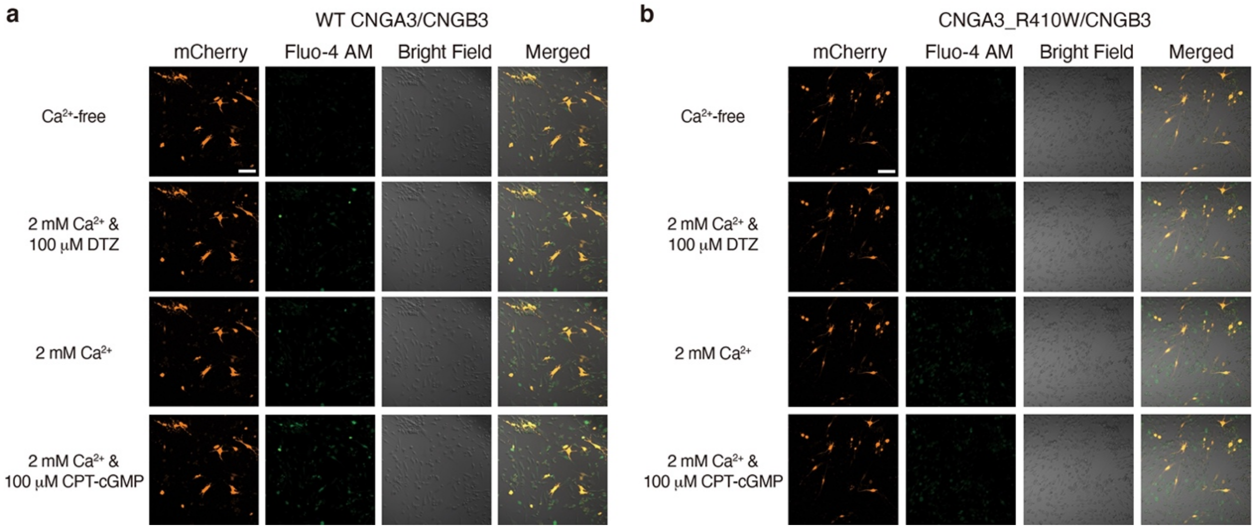

Supplementary Fig. 9 Ca^{2+} imaging of 661W cells transfected with WT or mutant CNGA3/CNGB3. Representative images of 661W cells transfected with WT (**a**) or mutant (**b**) CNGA3/CNGB3. mCherry was used as a normalization factor for cell number and transfection efficiency. Merged images show that most of the cells did not display mCherry signal and/or did not turn green upon the addition of 100 μM CPT-cGMP, indicating a low transfection efficiency. The images were selected from the view fields that had the highest numbers of transfected cells. Scale bars: 100 μm.

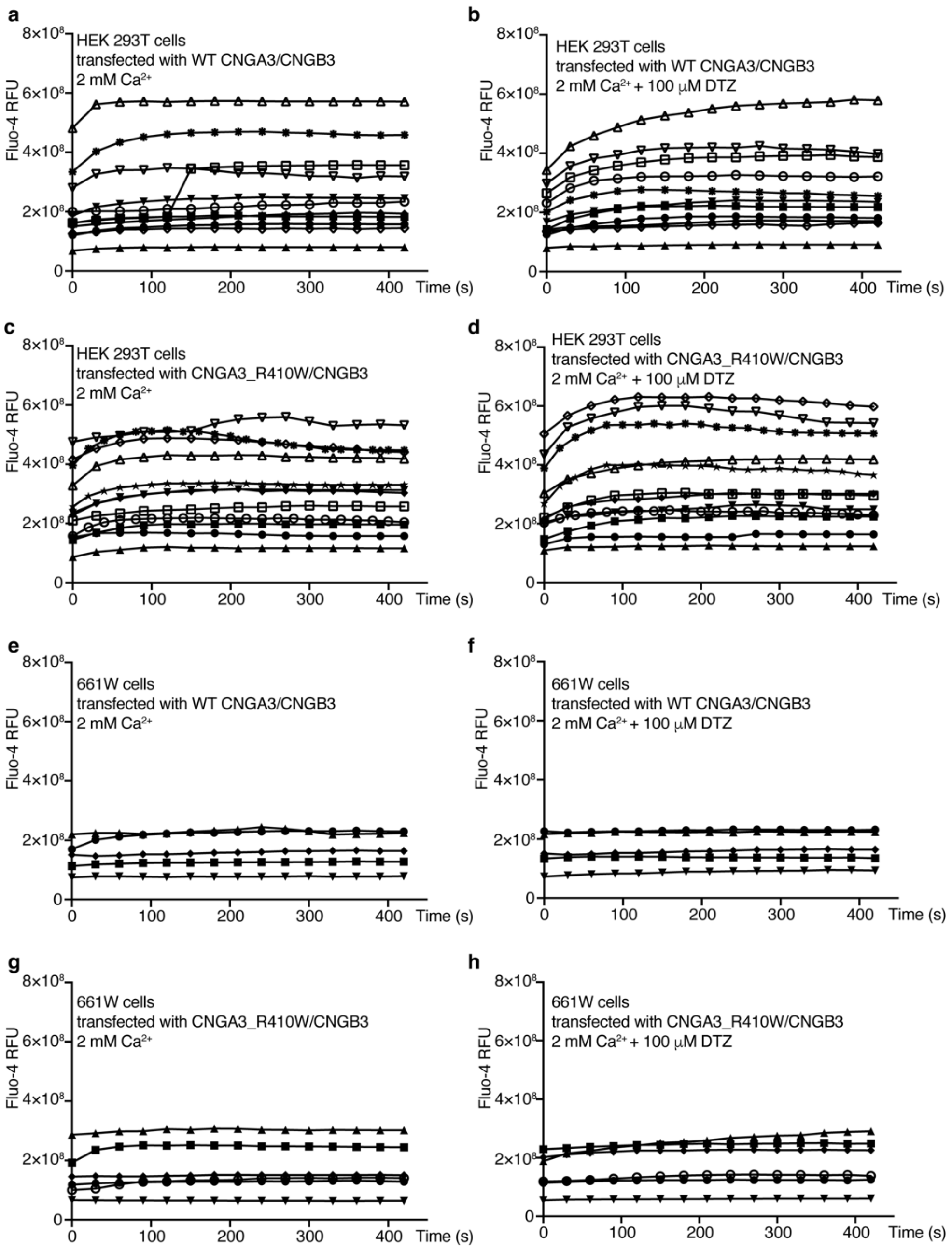

Supplementary Fig. 10 Temporal change of Fluo-4 fluorescence during Ca^{2+} imaging. Change of Fluo-4 relative fluorescent units (RFU) in HEK 293T cells (**a**-**d**) and 661W cells (**e**-**h**) transfected with the indicated channels under different imaging conditions. Each symbol represents a different well. Each point represents a snapshot taken at that time point. Each condition was imaged for 7 minutes and an image was taken every 20 or 30 seconds. The image with the highest RFU was selected for data analysis.

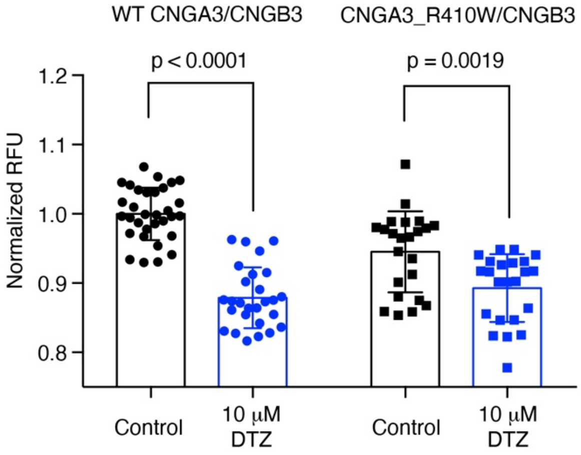

Supplementary Fig. 11 Long-term treatment with diltiazem is toxic to cells. Cell viability of HEK 293T cells expressing CNGA3_R410W/CNGB3 48 hours after transfection. Cells were continuously treated with 10 μM DTZ 6 hours after transfection. Data is presented as mean ± SD. Each point represents one well of cells from one of three cultures. Student's T-test was used to evaluate the statistical significance between groups.
