# Supplementary material for: Structural and functional characterization of an achromatopsia-associated mutation in a phototransduction channel
Source: Commun Biol. 2022 Mar 1;5:190. doi: 10.1038/s42003-022-03120-6 (PMC8888761; doi:10.1038/s42003-022-03120-6)

Fig. 5a Uncropped western blot gels

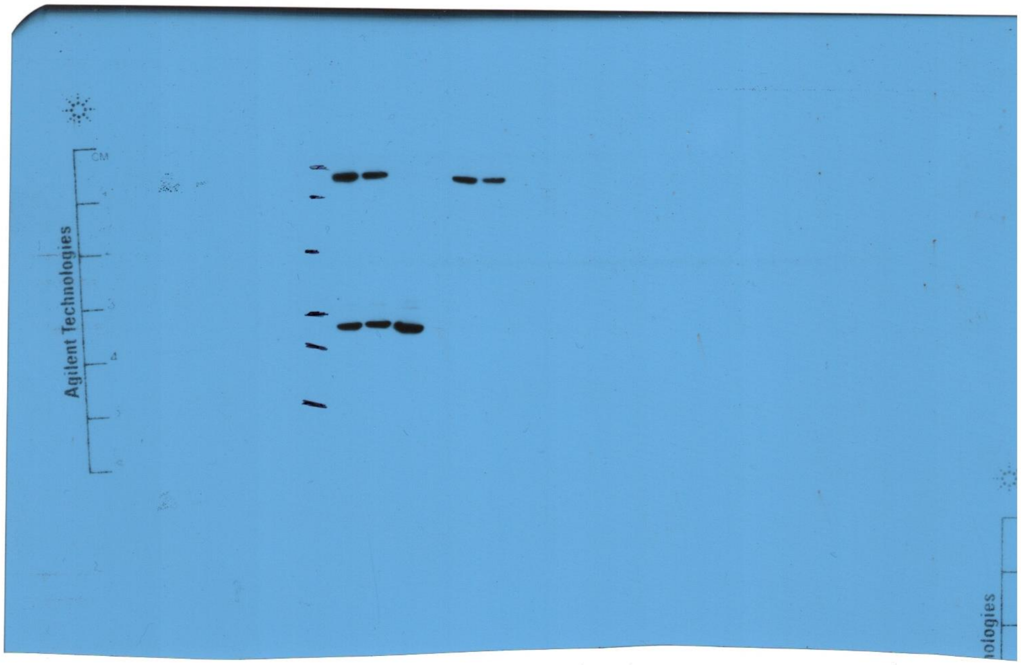

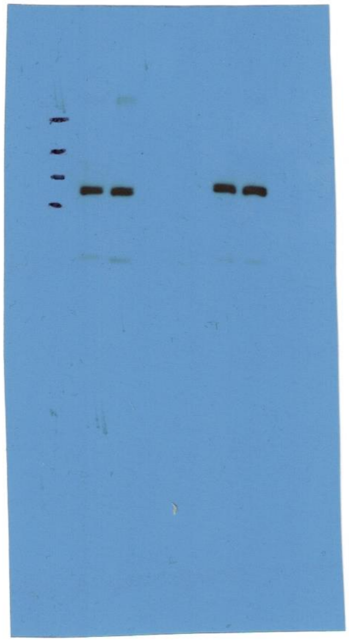

Supplementary Fig. 5a Uncropped SDS-PAGE gel

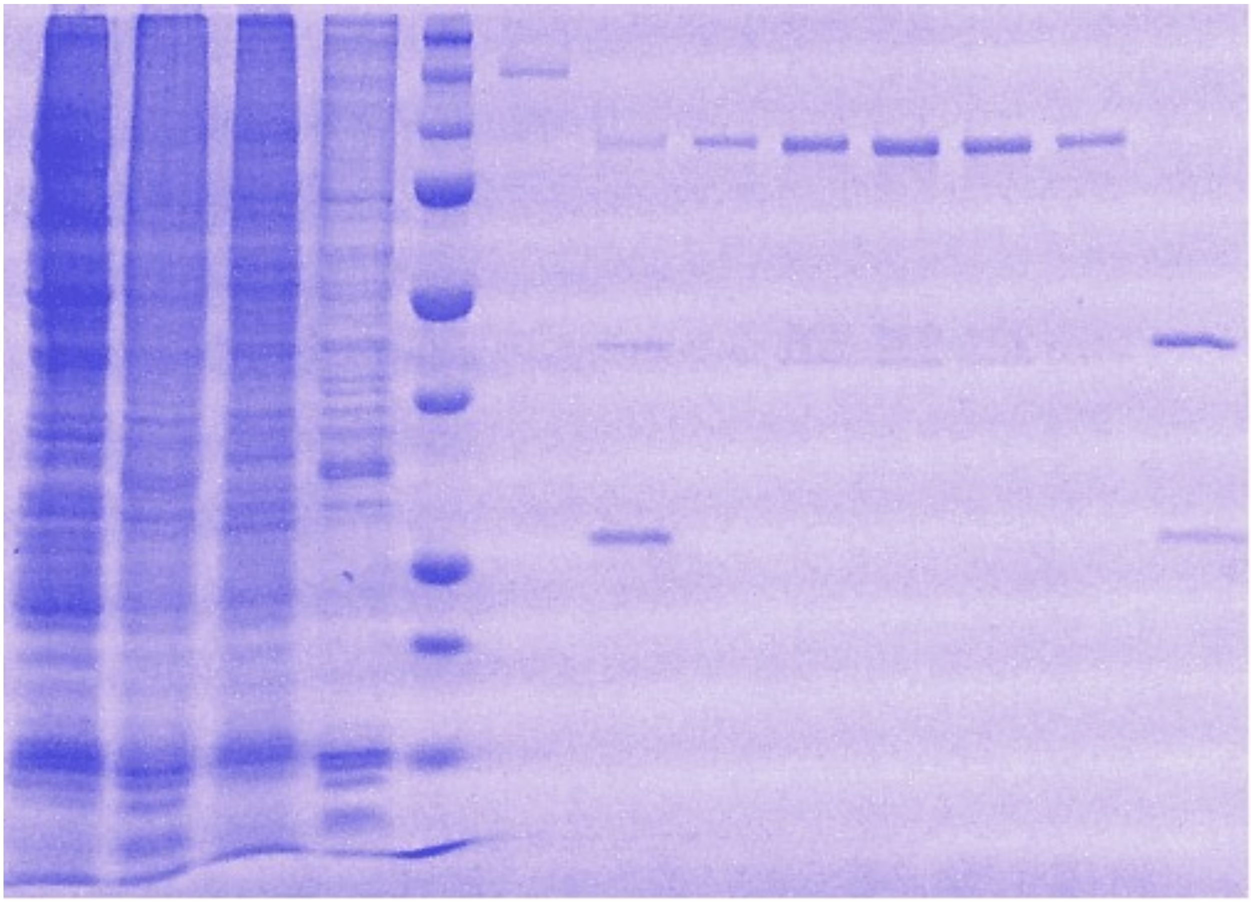

Supplementary Fig. 5b Uncropped SDS-PAGE gel

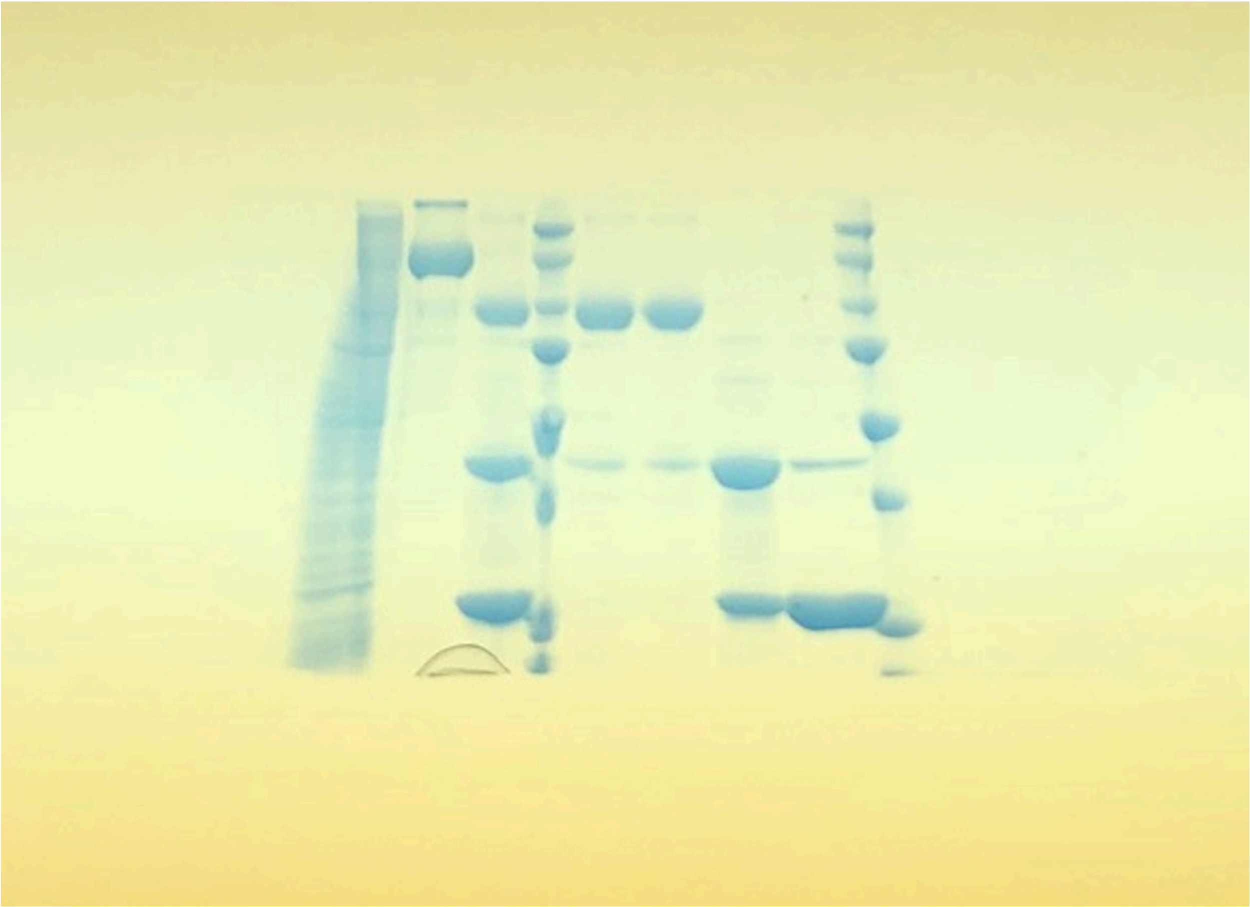

Supplementary Fig. 5c Uncropped SDS-PAGE gel

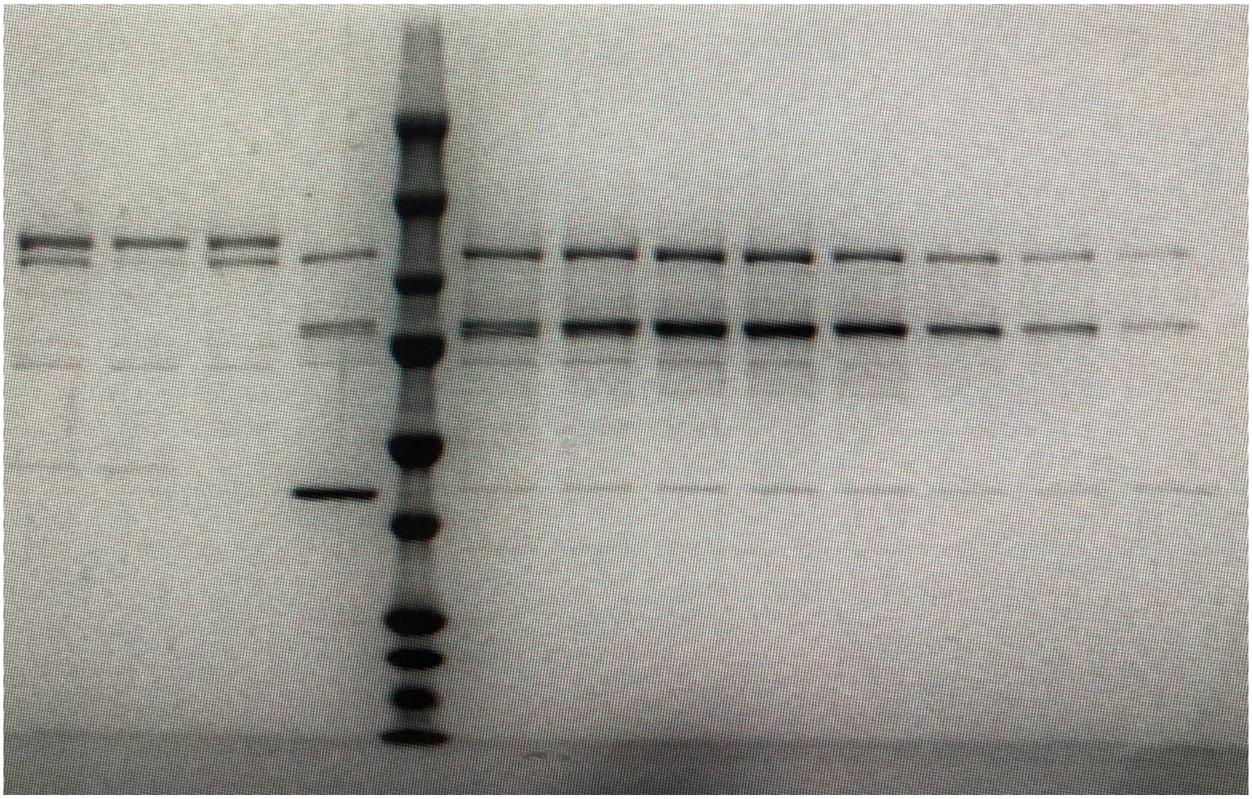

Supplementary Fig. 5d Uncropped SDS-PAGE gel

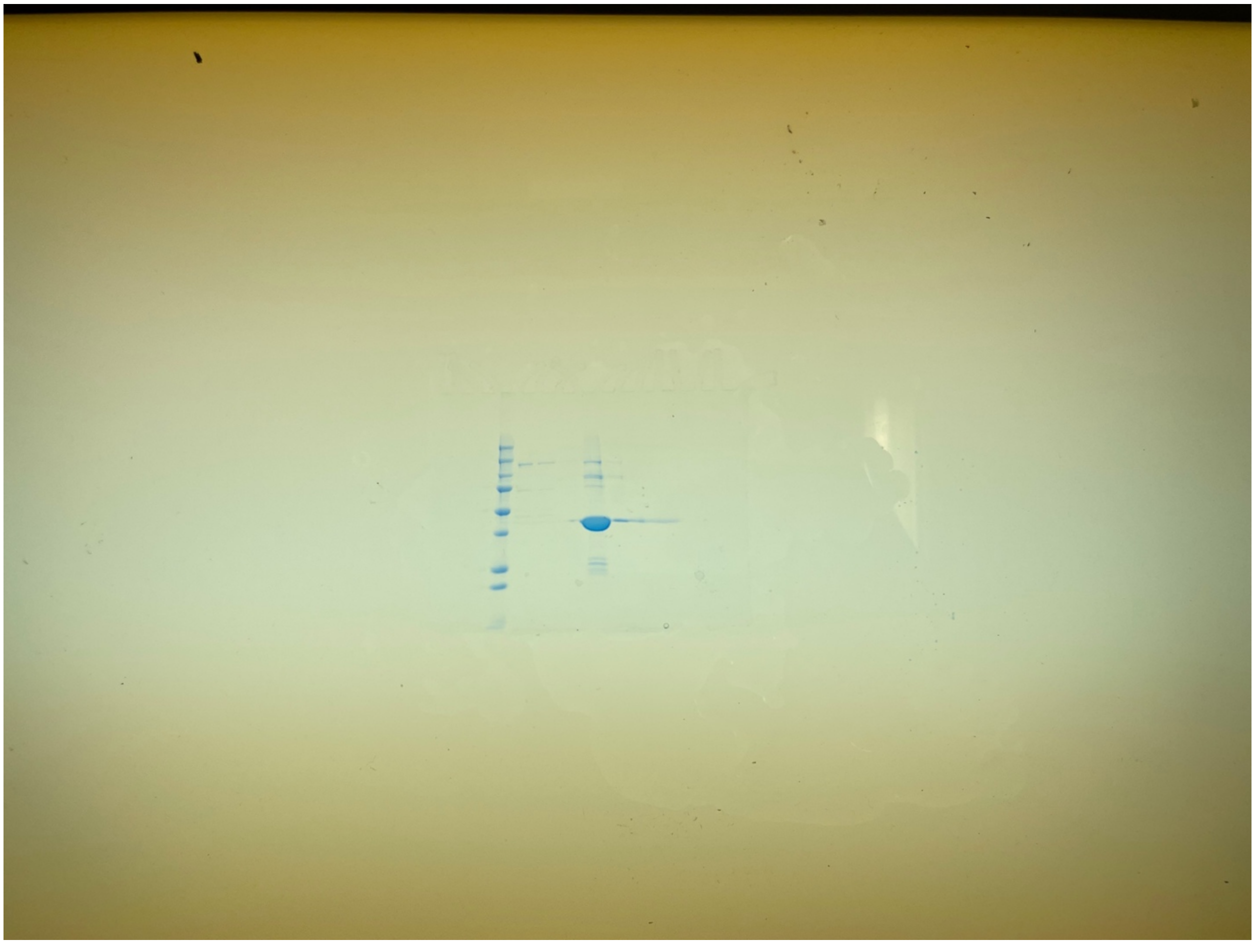

Supplement: Supplementary file 2 — Supplementary Data 1 [file 42003_2022_3120_MOESM2_ESM.pdf]
