# Supplementary material for: Structural and functional characterization of an achromatopsia-associated mutation in a phototransduction channel
Source: Commun Biol. 2022 Mar 1;5:190. doi: 10.1038/s42003-022-03120-6 (PMC8888761; doi:10.1038/s42003-022-03120-6)
Supplement: Supplementary file 4 — Reporting Summary [file 42003_2022_3120_MOESM4_ESM.pdf]

nature portfolio

Corresponding author(s): Jian Yang

Last updated by author(s): Jan 14, 2022

Reporting Summary

Nature Portfolio wishes to improve the reproducibility of the work that we publish. This form provides structure for consistency and transparency in reporting. For further information on Nature Portfolio policies, see our Editorial Policies and the Editorial Policy Checklist.

Statistics

For all statistical analyses, confirm that the following items are present in the figure legend, table legend, main text, or Methods section.

n/a	Confirmed	
☐	☒	The exact sample size (n) for each experimental group/condition, given as a discrete number and unit of measurement
☐	☒	A statement on whether measurements were taken from distinct samples or whether the same sample was measured repeatedly
☐	☒	The statistical test(s) used AND whether they are one- or two-sided<br>*Only common tests should be described solely by name; describe more complex techniques in the Methods section.*
☒	☐	A description of all covariates tested
☒	☐	A description of any assumptions or corrections, such as tests of normality and adjustment for multiple comparisons
☐	☒	A full description of the statistical parameters including central tendency (e.g. means) or other basic estimates (e.g. regression coefficient) AND variation (e.g. standard deviation) or associated estimates of uncertainty (e.g. confidence intervals)
☐	☒	For null hypothesis testing, the test statistic (e.g. F, t, r) with confidence intervals, effect sizes, degrees of freedom and P value noted<br>*Give P values as exact values whenever suitable.*
☒	☐	For Bayesian analysis, information on the choice of priors and Markov chain Monte Carlo settings
☒	☐	For hierarchical and complex designs, identification of the appropriate level for tests and full reporting of outcomes
☒	☐	Estimates of effect sizes (e.g. Cohen's d, Pearson's r), indicating how they were calculated

Our web collection on statistics for biologists contains articles on many of the points above.

Software and code

Policy information about availability of computer code

Data collection	LEGINON 3.5, pCLAMP 8.2
Data analysis	MotionCor2, GCTF v1.06, RELION 3.0.7, cryoSPARC 3.2.0, Microsoft Excel v15.26, UCSF Chimera 1.16, COOT 0.9 EL, Phenix-1.15.2-3472, PyMol 2.3.2, MolProbity 4.2, Prism 7

For manuscripts utilizing custom algorithms or software that are central to the research but not yet described in published literature, software must be made available to editors and reviewers. We strongly encourage code deposition in a community repository (e.g. GitHub). See the Nature Portfolio guidelines for submitting code & software for further information.

Data

Policy information about availability of data

All manuscripts must include a data availability statement. This statement should provide the following information, where applicable:

- Accession codes, unique identifiers, or web links for publicly available datasets
- A description of any restrictions on data availability
- For clinical datasets or third party data, please ensure that the statement adheres to our policy

The data supporting the findings of this study are available within the paper. The cryo-EM density maps for cGMP-bound open state, apo open state, and apo closed state of TAX-4_R421W have been deposited to the Electron Microscopy Data Bank under accession codes EMD-24113, EMD-24115 and EMD-24114, respectively. The corresponding atomic coordinates have been deposited to the Protein Data Bank under accession codes 7N15, 7N17 and 7N16, respectively. Uncropped western blot and SDS-PAGE gels are provided in Supplementary Data 1. Source data for plotting and statistics are provided in Supplementary Data 2. All other data including cryo-EM density maps in Supplementary Fig. 6 are available from the corresponding author on reasonable request.

Field-specific reporting

Please select the one below that is the best fit for your research. If you are not sure, read the appropriate sections before making your selection.

☒ Life sciences ☐ Behavioural & social sciences ☐ Ecological, evolutionary & environmental sciences

For a reference copy of the document with all sections, see nature.com/documents/nr-reporting-summary-flat.pdf

Life sciences study design

All studies must disclose on these points even when the disclosure is negative.

Sample size	For most functional studies, the sample sizes was >5 to ensure adequate power.
Data exclusions	No data were excluded from the analysis.
Replication	We did not replicate the structural experiments, which are standard practice. The electrophysiological experiments, intracellular calcium imaging and cell viability assays were readily and reliably reproduced.
Randomization	Randomization is not relevant to the structural experiments because no group allocation was performed. For electrophysiological recordings, intracellular calcium imaging, and cell viability assays, experiments with different conditions (e.g., WT vs. mutant channels) were performed in an alternating order on the same day.
Blinding	Blinding was not performed for structural experiments as subjective analysis was not needed and no group allocation was performed. In liposome recordings, the experiments were not randomized, and the investigators were not blinded to allocation during experiments and outcome assessment. Randomized and blind experiments were conducted in whole-cell recordings of WT and mutant TAX-4 and CNGA3/CNGB3 channels, and in intracellular calcium imaging and cell viability assays of WT and mutant CNGA3/CNGB3 channels.

Reporting for specific materials, systems and methods

We require information from authors about some types of materials, experimental systems and methods used in many studies. Here, indicate whether each material, system or method listed is relevant to your study. If you are not sure if a list item applies to your research, read the appropriate section before selecting a response.

Materials & experimental systems

n/a	Involved in the study	
☒	☐	Antibodies
☐	☒	Eukaryotic cell lines
☒	☐	Palaeontology and archaeology
☒	☐	Animals and other organisms
☒	☐	Human research participants
☒	☐	Clinical data
☒	☐	Dual use research of concern

Methods

n/a	Involved in the study	
☒	☐	ChIP-seq
☒	☐	Flow cytometry
☒	☐	MRI-based neuroimaging

Eukaryotic cell lines

Policy information about cell lines

Cell line source(s)	SF9 cells from Invitrogen.<br>HEK 293T and HEK 293S GnTi- cells from American Type Culture Collection (ATCC).
Authentication	Cell lines were directly purchased from Invitrogen and ATCC. Cell line authentication was not performed.
Mycoplasma contamination	Cell lines were not tested for mycoplasma contamination.
Commonly misidentified lines<br>(See ICLAC register)	No commonly misidentified cell lines were used in the study.
